# Multidimensional assessment of adverse events of finasteride：a real-world pharmacovigilance analysis based on FDA Adverse Event Reporting System (FAERS) from 2004 to April 2024

**DOI:** 10.1371/journal.pone.0309849

**Published:** 2025-03-24

**Authors:** Xiaoling Zhong, Yihan Yang, Sheng Wei, Yuchen Liu

**Affiliations:** 1 Department of Acupuncture and Moxibustion, Shenzhen Luohu District Hospital of Chinese Medicine, Shenzhen, China,; 2 Department of Acupuncture, Guangdong Provincial Hospital of Traditional Chinese Medicine, Guangzhou, China; 3 The Institution of Rehabilitation Industry, Fujian University of Traditional Chinese Medicine, Fuzhou, China; 4 Department of General Medicine, The Second Affiliated Hospital of Wannan Medical College, Wuhu, China; Ladoke Akintola University of Technology, NIGERIA

## Abstract

**Background:**

Finasteride is commonly utilized in clinical practice for treating androgenetic alopecia, but real-world data regarding the long-term safety of its 0adverse events(AEs) remains incomplete, necessitating ongoing supplementation. This study aims to evaluate the AEs associated with finasteride use, based on data from the US Food and Drug Administration Adverse Event Reporting System (FAERS), to contribute to its safety assessment.

**Methods:**

We reviewed AE reports associated with finasteride from the US Food and Drug Administration Adverse Event Reporting System database, covering the period from the first quarter of 2004 to the first quarter of 2024. We assessed the safety of finasteride medication and AEs using four proportional disproportionality analyses: reported odds ratio (ROR), proportionate reporting ratio (PRR), Bayesian Confidence Propagation Neural Network (BCPN), and Multi-Item Gamma Poisson Shrinkage (MGPS). These methods were used to evaluate whether there is a significant association between finasteride drug use and AEs. To investigate potential safety issues related to drug use, we further analyzed the similarities and differences in the onset time and AEs by sex, as well as the similarities and differences in AEs by age.

**Results:**

A total of 11,557 AE reports in which finasteride was the primary suspected drug were analysed. The majority of patients were male (86.04%) and a significant proportion were young adults aged 18-45 years (27.22%). A total of 73 different AEs were categorised into 7 system organ classes (SOCs), with common AEs including erectile dysfunction and sexual dysfunction. In addition, we identified previously unlisted AEs, including Peyronie’s disease and post-5α reductase inhibitor syndrome. Of the reported AEs, 102 occurred in men and 7 in women, with depression and anxiety being significant AEs observed in both sexes. When analysed by age group, there were 17 AEs in patients aged ≤ 18 years, 157 in patients aged 18-65 years and 133 in patients aged ≥ 65 years. Common AEs in all age groups included erectile dysfunction, decreased libido, depression, suicidal ideation, psychotic disturbances and attention disorders. The median time to onset of all AEs was 61 days, with the majority occurring within the first month of treatment. Notably, a significant number of AEs persisted beyond one year of treatment.

**Conclusion:**

The results of our study uncovered both known and novel AEs associated with finasteride medication. Some of these AEs were identical to the specification, and some of them signaled AEs that were not demonstrated in the specification. In addition, some AEs showed variations based on sex and age in our study. Consequently, our findings offer valuable insights for future research on the safety of finasteride medication and are anticipated to enhance its safe use in clinical practice.

## Introduction

Androgenetic alopecia (AGA), commonly referred to as male-pattern baldness, is characterized by hair loss driven by dihydrotestosterone, a potent derivative of testosterone [1]. It has a significant negative physiological and psychological impact on patients. Patients with AGA experience adverse effects such as sexual dysfunction, affective disorders, stigma and low self-esteem compared to normal individuals [2,3], especially in younger patients [4]. Fortunately, however, since receiving approval for treating AGA in 1997, finasteride has gained popularity for AGA treatment and has shown positive therapeutic outcomes [5].

Finasteride, as the main drug for the treatment of AGA, works by efficiently inhibiting 5-alpha reductase inhibitors (5-ARIs) [6]. Since the synthesis of sex hormones in the body is dependent on steroid reductases, and SRD5A2 is a biofilm chimeric steroid in the class of steroid reductase enzymes, SRD5A2 can synthesize dihydrotestosterone by catalyzing the reductive reaction of testosterone, which can lead to the onset of AGA if it is too high [7]. So finasteride as 5-alpha reductase inhibitor is the anti-androgenic drug that is used to treat both diseases and is widely available [8]. It is noteworthy that patients with Benign prostatic hyperplasia and AGA experience AEs of depression or suicide when neuroactive steroids have a reduced ability to control mood, behavior, and cognition [9–11]. The data show that between 1998 and 2008, an estimated 4.6 million men were treated with at least 1 mg of finasteride [12]. More importantly, as of June 2021, the FDA AE disclosure data have reported up to 10,295 serious AEs with finasteride use. Unfortunately, however, safety information regarding finasteride in patients with AGA primarily stems from clinical trials and post-marketing observational studies. The frequency of persistent AEs associated with it remains unclear, underscoring the necessity for additional evidence-based research to investigate the potential risks of finasteride use [13]. A Real-world study, with their large data samples and rigorous inclusion and exclusion criteria, have emerged as a useful tool to explore AEs and to compensate for clinical trials. An effective way of exploring adverse drug events and bridging the gap between clinical trials to provide us with evidence-based information.

FAERS is an openly available database created by the FDA that gathers instances of adverse drug events reported globally, with a large amount of real-world data and extensive geographic coverage of records of AEs from practicing physicians, pharmacists, registered nurses, consumers, and other healthcare professionals, among others [14,15]. Being the largest pharmacovigilance database globally, FAERS is an invaluable resource for identifying AEs associated with drug usage [16]. Thus, the objective of this study was to assess the long-term safety profile of finasteride following its market introduction using data extracted from FAERS database [17]. This study offers guidance and citations for the rational and safe clinical administration of finasteride dosage.

## Materials and methods

### Date sources

FAERS is a database for public post-marketing safety monitoring of drugs, and this retrospective analysis of adverse drug reactions utilized data sourced from the FAERS database. Our study gathered data from the FAERS Quarterly Data Extract Files spanning from Q1 2004 (FDA approval of finasteride) through Q1 2024.The FAERS database is updated quarterly and is widely recognized internationally for its large volume of data and standardization, and all the data within the FAERS database can be freely downloaded (https://open.fda.gov/data/downloads/).

### Data extraction

The FAERS data file contains seven comprehensive datasets: drug information (DRUG), information on adverse events (REAC), information on patient demographics and management (DEMO), information on patient outcomes (OUTC), information on the source of the report (RPSR), date of initiation and termination of medication (THER), and information on the indication of the drug (INDI). Whereas, the role of the reporting drug in each case was indicated in the DRUG table through specific codes: PS (primary suspect drug), SS (secondary suspect drug), C (concomitant drug), and I (interacting drug) [18]. Major endpoints were identified as disability (DS), hospitalisation (HO), life-threatening (LT), death (DE), and need for intervention to prevent permanent injury/damage (RI), congenital anomalies (CA), and other serious medical events (OT). In this study, cases were identified by utilizing drug names listed in the DRUG file, and selections were determined based on PS outcomes.

AEs in the FAERS database were categorized using the international medical dictionary MedDRA, which organizes terms into a structural hierarchy comprising preferred term (PT), system organ class (SOC), high level group term (HLGT), high level term (HLT), and lowest level term (LLT).We meticulously identified individual AE) reports linked to finasteride at the SOC and PT levels. Additionally, our study concentrated on analyzing the specific drugs documented in the drug files, namely finasteride (generic name) and proscar (trade name), with the aim of obtaining a standardization of the AEs associated with finasteride after a name change [19,20]. Finally, by means of the Medex UIMA 1.8.3 system the standardization of drug names [21,22].

### Data cleaning

A retrospective pharmacovigilance investigation was undertaken to assess the post-market safety of finasteride. Data were sourced from the openly accessible FAERS database, encompassing raw FAERS data related to finasteride spanning from the first quarter of 2004 to the first quarter of 2024. All data retrieved from the US FDA website were imported into R (Version 4.2.2) for subsequent analysis. Duplicate reports were identified and eliminated in accordance with FDA guidance, applying this rigorous methodology aimed at avoiding duplicate entry of AE reports and ensuring further analysis following a thorough evaluation of the safety profile of finasteride [23]. The operation was performed in the following manner, selecting the PRIMARYID, CASEID, and FDA_DT fields of the DEMO table and sorting the data by CASEID, FDA_DT, and PRIMARYID; when reports with the same identical CASEID are available, the report with the largest FDA_DT value is retained; when records with the same CASEID and FDA_DT are available, the record with the largest PRIMARYID value is retained [24]. Not only that, but the erroneous case reports noted on the FDA website were deleted as suggested, and the final obtained clinical characteristics of the patients who were administered finasteride were obtained Clinical characteristics including sex, age, reporting region, reporter, time of reporting, and outcome were the relevant AE data results.

### Statistical analysis

The four-grid proportional imbalance method, known for its widespread application in pharmacovigilance studies, was employed for the disproportionality analysis investigating potential associations between finasteride and all reported AEs [25], as documented in S1 Table. Reported dominance ratio (ROR), proportional reporting ratio (PRR), information component (IC) in Bayesian confidence propagation neural network (BCPNN), and multinomial gamma poisson shrinker (MGPS) are the four main specific metrics computed using standard formulas for evaluating potential correlations involving finasteride and AEs [26].

**Fig 1:**
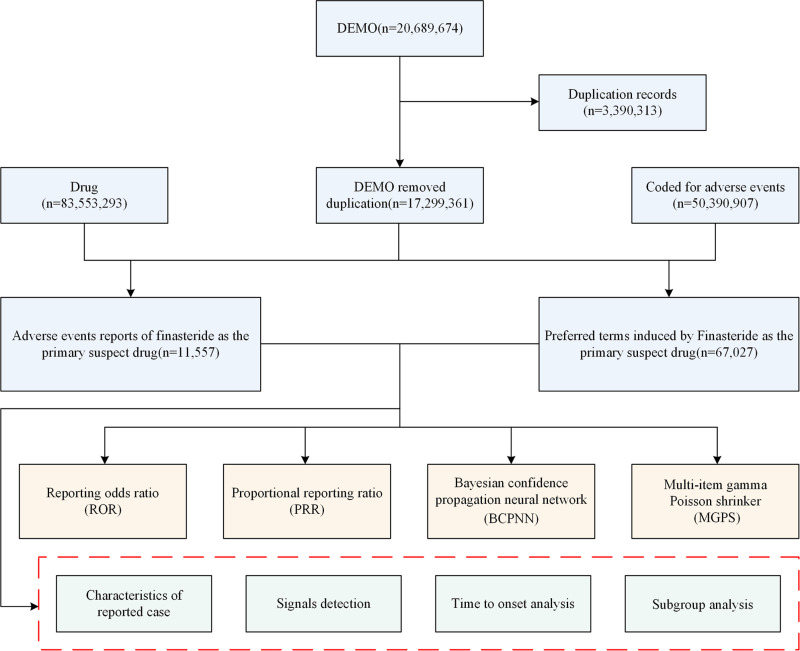
Flowchart depicting the selection of the study population.

In our study, the signals of the 4 AE recordings of the target drug were calculated, and a positive drug-associated AE signal was considered if it met at least one of the four algorithms (lower limit of 95% CI >  1, N ≥  3; PRR ≥  2, χ2 ≥  4, N ≥  3; IC025 >  0 or EBGM05 >  2) [27,28]. The methodologies and thresholds for the AE signals are detailed in S2 Table, while the procedural flowchart is depicted in Fig 1.

The flow diagram of selecting finasteride therapy related AEs from the FAERS database. DEMO, demographic and administrative information; FAERS, US Food and Drug Administration Adverse Event Reporting System.

### Time to onset (TTO) analysis

The onset time of AEs and the likelihood of a severe outcome were computed, with time to onset defined as the interval between EVENT_DT (date of AE onset) and START_DT (time of medication initiation), and median and interquartile ranges were used to describe the time to onset. In addition, AEs attributable to the route of administration were counted, calculated as number of serious outcomes/total number of events reported.

### Visualization of data

Images were produced using the ggplot2 package and GraphPad Prism 8.0.1. We employed a global heat map to depict data from countries that submitted reports (https://cran.r-project.org/web/packages/mapdata/index.html). A line graph was utilized to illustrate the trend in case numbers from the first quarter of 2004 to the first quarter of 2024. Additionally, to ascertain if the AE signal differed between males and females following finasteride administration, we created a volcano plot displaying log2-transformed PRP values on the x-axis and -log10-transformed adjusted P-values on the y-axis [24]. When PRP exceeded 1 and P.adj was greater than 0.05, it suggested a distinct AE signal between female and male patients. The demographic ratios of reported cases by sex and age, along with annual case counts, were processed and visualized using Excel tables.

### Ethics statement

The study involving human participants did not require ethical review and approval in compliance with local laws and institutional guidelines. Written informed consent from participants or their legally authorized representatives was not necessary for participation in this study in accordance with national regulations and institutional policies.

## Results

### Characteristics of reported case

**Fig 2:**
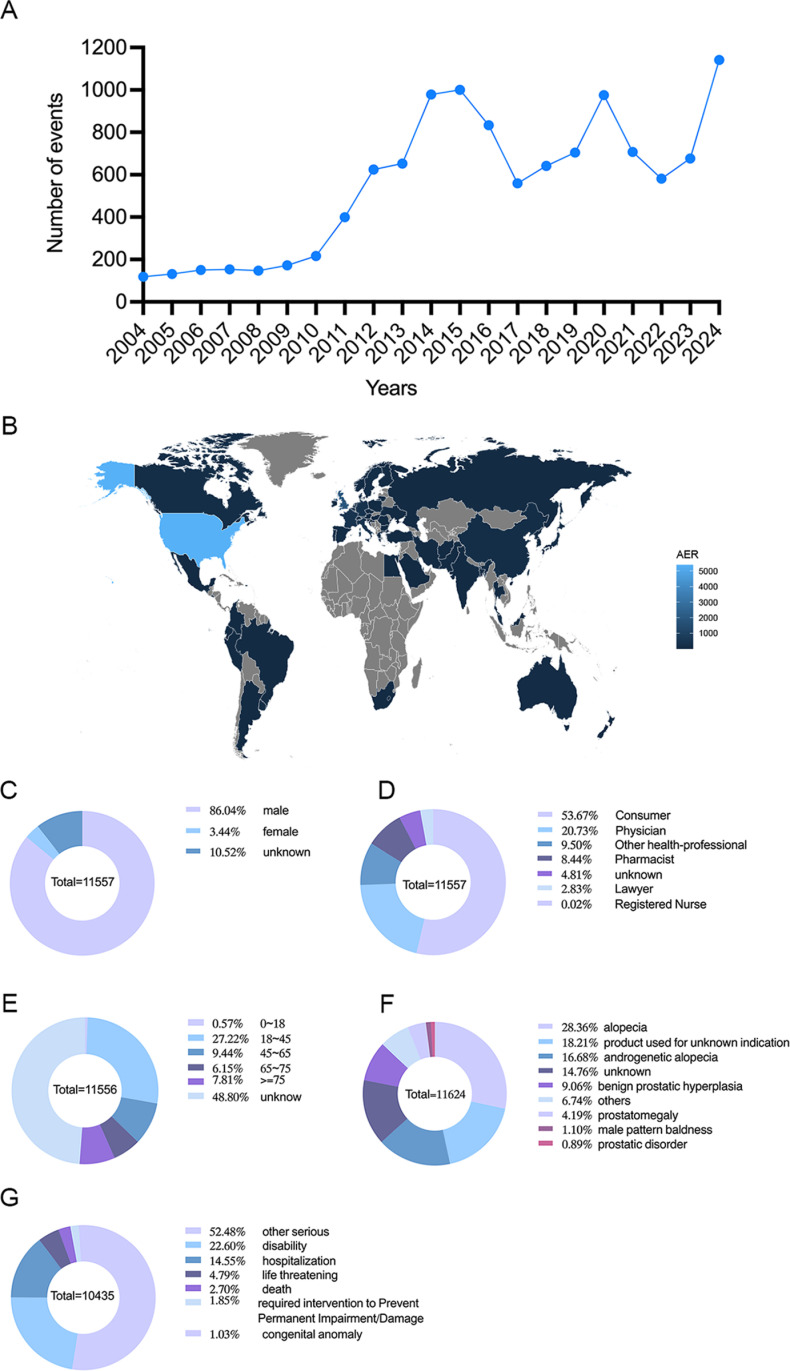
Basic information and patient characteristics according to the report. (A) The annual distribution of finasteride administration related AEs reports from 2004 to April 2024. (B) Country distribution of AEs for finasteride administration, brighter colors represent a higher number of reports, Map created in R using mapdata package (https://cran.r-project.org/web/packages/mapdata/index.html) and the FAERS database (https://open.fda.gov/data/downloads/). (C) Sex ratio of male and female in reported events. (D) Occupational information ratio in reported events. (E) Age distribution ratio in reported events. (F) Ratio of indications in reported events. (G) Ratio of outcomes in reported events. Visualization through proportional area map, larger areas represent more reporters.

From the first quarter of 2004 to the first quarter of 2024, a total of 20,689,674 adverse reaction reports were submitted, of which 11,557 were specifically related to finasteride use. Table 1 presents comprehensive details of the reports, encompassing patient demographics such as sex and age, the reporting year, the occupation of the reporter, and the country from which the report was submitted. The peak number of finasteride-related reports occurred in 2024 (1,141 cases, 9.87%), followed by 2015 (1,000 cases, 8.65%) (Fig 2A). Geographical information was not available for 27.44% of the reports, limiting insight into regional trends (Fig 2B). Reports were predominantly from the United States (46.68%), United Kingdom (16.19%), Italy (3.51%), France (3.35%) and Germany (2.83%). The majority of reports involved male patients (86.04%), 3.44% were female and 10.52% were of unknown sex (Fig 2C). The majority of reports were submitted by consumers (53.67%) and physicians (20.73%) (Fig 2D). In terms of age, the largest group of patients were aged 18-45 years (27.22%), followed by those aged 45-65 years (9.44%), and 7.81% were ≥ 75 years, with 48.80% of the age information being unknown (Fig 2E). The most commonly reported indications for finasteride use were alopecia (28.36%), androgenetic alopecia (16.68%) and benign prostatic hyperplasia (9.06%) (Fig 2F). Final patient outcomes included disability in 22.60% of cases and hospitalisation in 14.55% of cases (Fig 2G).

**Table 1:** Clinical characteristics of reports with finasteride as the primary suspected drug from the FAERS database.

Variable	Total(%)
**Year**	
2004	118(1.02)
2005	131(1.13)
2006	150(1.30)
2007	153(1.32)
2008	147(1.27)
2009	172(1.49)
2010	216(1.87)
2011	399(3.45)
2012	624(5.40)
2013	652(5.64)
2014	978(8.46)
2015	1000(8.65)
2016	833(7.21)
2017	559(4.84)
2018	641(5.55)
2019	704(6.09)
2020	975(8.44)
2021	707(6.12)
2022	581(5.03)
2023	676(5.85)
2024	1141(9.87)
**Sex**	
female	397(3.44)
male	9944(86.04)
unknown	1216(10.52)
**Age_yr**	41.00(29.00,67.00)
**Age_yrQ**	
0 ~ 18	66(0.57)
18 ~ 45	3146(27.22)
45 ~ 65	1091(9.44)
65 ~ 75	711(6.15)
>=75	903(7.81)
Unknown	5639(48.80)
**Reporter**	
Consumer	6203(53.67)
Physician	2396(20.73)
Other health-professional	1098(9.50)
Pharmacist	975(8.44)
unknown	556(4.81)
Lawyer	327(2.83)
Registered Nurse	2(0.02)
**Reported countries**	
United States	5395(46.68)
other	3171(27.44)
United Kingdom	1871(16.19)
Italy	406(3.51)
France	387(3.35)
Germany	327(2.83)
**Route**	
oral	7513(65.01)
other	3956(34.23)
transplacental	59(0.51)
buccal	15(0.13)
topical	14(0.12)
**Outcomes**	
other serious	5476(52.48)
disability	2358(22.60)
hospitalization	1518(14.55)
life threatening	500(4.79)
death	282(2.70)
required intervention to Prevent Permanent Impairment/Damage	193(1.85)
congenital anomaly	108(1.03)
**Time to onset**	61.00(6.00,463.75)
**Time to onsetQ**	
0 ~ 31	1337(19.05)
31 ~ 61	260(3.70)
61 ~ 91	163(2.32)
91 ~ 121	102(1.45)
121 ~ 150	80(1.14)
151 ~ 181	71(1.01)
181 ~ 361	278(3.96)
>=361	923(13.15)
unknow	3805(54.21)
**Indications**	
alopecia	3297(28.36)
androgenetic alopecia	1939(16.68)
benign prostatic hyperplasia	1053(9.06)
male pattern baldness	128(1.10)
others	784(6.74)
product used for unknown indication	2117(18.21)
prostatic disorder	103(0.89)
prostatomegaly	487(4.19)
unknown	1716(14.76)

### Signals detection based on SOC levels

**Fig 3:**
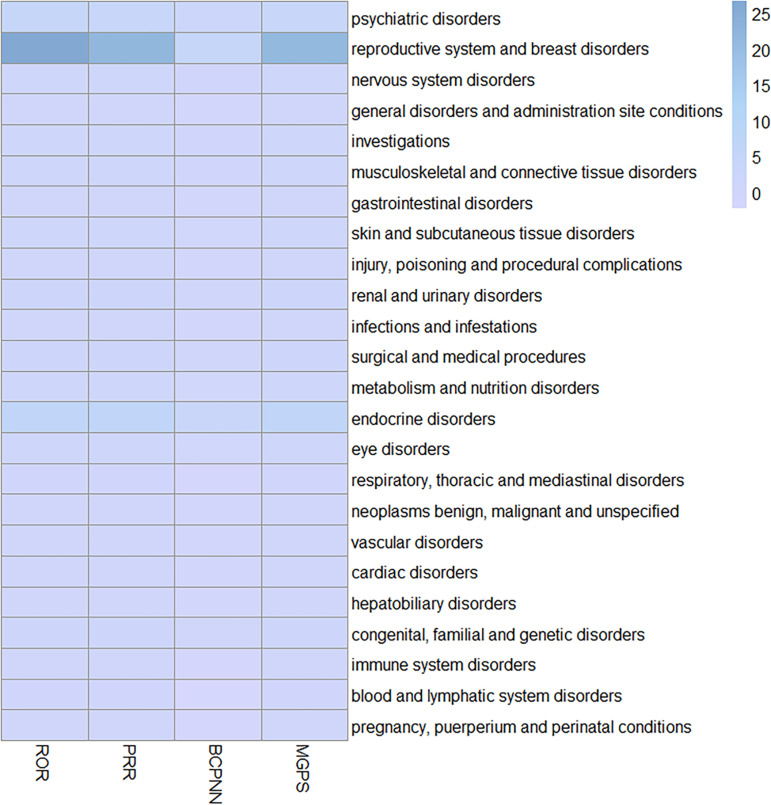
Signal strength of reports of finasteride administration at the SOC level.

Statistical analysis of finasteride dose-related SOCs showed that 24 were affected (S3 Table, Fig 3). Among these, reproductive and breast disorders, endocrine disorders, and psychiatric disorders showed the highest associations based on PRR ≥ 2, χ2 ≥ 4, and N ≥ 3. Specifically, reproductive and breast disorders had the strongest association (ROR = 26.68, PRR = 21.97, χ2 = 241136.36, IC = 4.42, EBGM = 21. 37), followed by endocrine disorders (ROR = 6.58, PRR = 6.49, χ2 = 5179.48, IC = 2.69, EBGM = 6.44) and psychiatric disorders (ROR = 4.57, PRR = 3.77, χ2 = 32212.4, IC = 1.91, EBGM = 3.76). When ranked by number of cases, the most commonly reported disorders were psychiatric (n = 14951), reproductive and breast (n = 12293), neurological (n = 5992), renal and urological (n = 1841), surgical and medical manipulation (n = 1268), endocrine (n = 1122), and various congenital familial genetic disorders (n = 282). These findings highlight key areas where finasteride-related AEs are commonly observed.

SOC distribution of adverse reactions associated with finasteride administration, with darker colours representing stronger signals.

Analysis of adverse reactions showed a strong correspondence with the SOCs listed in the drug insert, confirming the reliability of the data. The SOCs for which adverse reactions appeared in the drug insert included skin and subcutaneous tissue-like disorders (n=2561, ROR=0.68, PRR=0.69, χ2=369.34, IC=-0.53, EBGM=0.69) and neoplasms benign, malignant and unspecified (n=1047, ROR=0.56, PRR=0.56, χ2=364.75, IC=-0.83, EBGM=0.56). However, these signals did not meet positive thresholds across all four signal intensities, suggesting the need for further investigation.

### Signals detection based on preferred term levels

In our pharmacovigilance analysis, we identified 73 AEs categorised at the PT level in Table 2. We focused on PTs with more than 100 cases, as this is often a strong indication of a drug-related AE [29]. Key findings include reproductive system and breast disorders such as erectile dysfunction (n = 3377), sexual dysfunction (n = 2160) and ejaculatory dysfunction (n = 652), and psychiatric disorders such as decreased sexual desire (n = 1476). Notably, endocrine disorders such as hypogonadism (n = 302) and post-5α-reductase inhibitor syndrome (n = 166) were also identified, although these are not listed in the drug specifications and warrant further attention.

**Table 2:** Signal strength of reports of finasteride administration at the preferred term level in the FAERS database.

PTs and categories	Case Reports	ROR(95% CI)	PRR(95% CI)	chisq	IC(IC025)	EBGM(EBGM05)
**Reproductive system and breast disorders**						
Erectile dysfunction	3377	144.15(138.85, 149.66)	136.94(131.68, 142.41)	385588.12	6.86(6.8)	115.97(112.39)
Sexual dysfunction	2160	220.84(210.4, 231.79)	213.75(205.53, 222.3)	356099.85	7.38(7.31)	166.6(159.99)
Ejaculation disorder	652	243.38(222.76, 265.92)	241.02(222.85, 260.68)	117980.64	7.51(7.39)	182.7(169.65)
Testicular pain	618	223(203.8, 244)	220.95(200.32, 243.7)	104552.68	7.42(7.29)	170.94(158.54)
Ejaculation failure	487	279.44(251.79, 310.14)	277.42(251.52, 305.98)	97945.39	7.66(7.52)	202.84(185.9)
Penile size reduced	398	662.12(578.62, 757.67)	658.19(573.81, 754.98)	139167.42	8.46(8.28)	351.19(313.73)
Genital hypoaesthesia	235	337.89(289.65, 394.18)	336.71(287.84, 393.87)	54305.2	7.86(7.65)	232.77(204.62)
Organic erectile dysfunction	218	1094.72(889.08, 1347.92)	1091.16(879.54, 1353.7)	96782.38	8.8(8.56)	445.36(374.2)
Peyronie’s disease	215	286.11(244.49, 334.82)	285.2(243.81, 333.62)	44127.17	7.69(7.48)	206.96(181.45)
Testicular atrophy	213	301.56(257.2, 353.56)	300.6(256.98, 351.63)	45419.19	7.75(7.53)	214.94(188.15)
Testicular disorder	158	157.28(132.48, 186.73)	156.91(131.53, 187.18)	20246.36	7.02(6.78)	129.96(112.58)
Painful ejaculation	96	707.65(535.46, 935.2)	706.64(537.07, 929.76)	34847.63	8.51(8.16)	364.51(288.66)
Varicocele	89	461.45(354.37, 600.89)	460.84(357.18, 594.58)	25304.84	8.16(7.81)	285.94(229.26)
Male sexual dysfunction	74	136.32(106.41, 174.65)	136.17(105.54, 175.69)	8405.1	6.85(6.5)	115.42(93.81)
Prostatic pain	68	173.83(133.52, 226.32)	173.65(134.59, 224.04)	9480.26	7.14(6.77)	141.22(113.25)
Infertility male	64	136.25(104.39, 177.84)	136.12(103.46, 179.1)	7266.87	6.85(6.47)	115.38(92.33)
Penile curvature	63	328.78(244.5, 442.11)	328.48(244.81, 440.75)	14308.32	7.84(7.43)	228.81(178.59)
Male genital atrophy	52	1116.34(727.21, 1713.69)	1115.47(724.75, 1716.82)	23293.43	8.81(8.32)	449.35(313.93)
Genital atrophy	49	944(619.84, 1437.69)	943.31(625.03, 1423.67)	20441.53	8.71(8.21)	418.62(294.41)
Epididymal cyst	49	800.35(535.17, 1196.92)	799.77(529.92, 1207.04)	18928.1	8.6(8.11)	387.77(276.9)
Genital paraesthesia	49	230.1(167.07, 316.91)	229.93(168.04, 314.62)	8550.4	7.46(7.02)	176.26(134.84)
Scrotal disorder	38	115.57(82.13, 162.64)	115.51(82.78, 161.19)	3738.49	6.65(6.16)	100.24(75.32)
Male reproductive tract disorder	26	330.99(208.65, 525.06)	330.86(206.71, 529.59)	5935.04	7.85(7.22)	229.96(156.31)
Oligospermia	23	239.92(150.02, 383.68)	239.84(149.84, 383.9)	4145.99	7.51(6.86)	182.01(122.88)
Spermatocele	22	660.92(372.64, 1172.21)	660.7(374.24, 1166.43)	7708.26	8.46(7.74)	351.91(217.87)
Testicular cyst	21	133.66(84.02, 212.63)	133.62(83.48, 213.88)	2346.52	6.83(6.18)	113.58(77.02)
Penile vascular disorder	17	170.22(100.54, 288.2)	170.18(100.25, 288.89)	2330.86	7.12(6.39)	138.92(89.42)
Breast disorder male	17	138.77(82.71, 232.83)	138.73(83.34, 230.93)	1962.06	6.87(6.15)	117.25(76.05)
Hypospermia	16	750.98(375.54, 1501.75)	750.8(378.09, 1490.91)	5990.42	8.55(7.71)	375.9(210.49)
Feminisation acquired	16	387.6(212, 708.66)	387.51(211.06, 711.48)	4068.39	8(7.2)	255.93(154.48)
Prostatic calcification	16	130.6(76.8, 222.1)	130.57(76.92, 221.65)	1752.52	6.8(6.06)	111.38(71.43)
Genital disorder	13	157.46(86.59, 286.31)	157.43(85.75, 289.04)	1670.38	7.03(6.2)	130.31(79.01)
Testis discomfort	13	131.92(73.17, 237.86)	131.9(73.26, 237.47)	1436.42	6.81(6)	112.34(68.6)
Penile discomfort	12	121.77(66.17, 224.12)	121.75(66.31, 223.54)	1236.59	6.71(5.87)	104.9(62.97)
Teratospermia	10	208.59(103.51, 420.32)	208.56(102.99, 422.35)	1616.56	7.35(6.41)	163.43(90.94)
Prostate tenderness	9	168.95(81.98, 348.2)	168.93(81.8, 348.86)	1226.47	7.11(6.13)	138.09(75.4)
Testicular microlithiasis	6	250.29(99.35, 630.55)	250.27(99.62, 628.75)	1117.22	7.55(6.34)	187.95(86.75)
Prostatic atrophy	5	250.29(90.96, 688.68)	250.27(90.32, 693.49)	931.02	7.55(6.25)	187.95(80.58)
Epididymal disorder	4	176.67(59.44, 525.06)	176.66(58.95, 529.44)	565.58	7.16(5.76)	143.2(57.56)
Epididymal tenderness	3	450.5(107.66, 1885.15)	450.48(107.72, 1883.93)	840.9	8.14(6.44)	281.93(85.11)
Oligoasthenozoospermia	3	160.89(46.24, 559.89)	160.89(45.89, 564.04)	392.56	7.05(5.49)	132.67(46.73)
**Renal and urinary disorders**						
Terminal dribbling	13	278.92(147.57, 527.2)	278.87(148.94, 522.15)	2624.52	7.67(6.81)	203.61(119.52)
Post micturition dribble	6	136.52(57.2, 325.83)	136.51(57.63, 323.37)	682.93	6.85(5.7)	115.66(55.86)
**Endocrine disorders**						
Hypogonadism	302	365.59(318.58, 419.54)	363.95(317.29, 417.47)	73623.19	7.94(7.75)	245.45(218.75)
Post 5-alpha-reductase inhibitor syndrome	166	10411.85(5795.02, 18706.87)	10386.07(5768.81, 18698.91)	116208.64	9.45(9.15)	701.12(429.4)
Hypogonadism male	79	418.19(317.58, 550.67)	417.7(317.46, 549.59)	21101.15	8.07(7.7)	268.74(213.47)
Androgen deficiency	77	238.18(184.31, 307.8)	237.91(184.4, 306.95)	13794.31	7.5(7.14)	180.9(145.97)
Testicular failure	72	1061.09(741.19, 1519.07)	1059.95(744.85, 1508.36)	31583.88	8.78(8.36)	440.08(325.95)
Secondary hypogonadism	42	137.19(98.73, 190.63)	137.1(98.25, 191.31)	4798.42	6.86(6.39)	116.09(88.15)
Oestrogenic effect	4	333.71(102.76, 1083.67)	333.69(102.95, 1081.62)	918.53	7.85(6.38)	231.32(86.34)
**Psychiatric disorders**						
Libido decreased	1476	119.46(113.02, 126.26)	116.85(110.18, 123.93)	146728.83	6.66(6.58)	101.25(96.66)
Loss of libido	1301	160.75(151.35, 170.73)	157.65(148.65, 167.2)	167389.74	7.03(6.94)	130.46(124.05)
Orgasm abnormal	109	135.26(110.3, 165.88)	135.04(111, 164.28)	12291.97	6.84(6.55)	114.61(96.62)
Male orgasmic disorder	108	240.29(193.46, 298.45)	239.9(193.37, 297.62)	19472.09	7.51(7.21)	182.05(151.85)
Premature ejaculation	83	124.29(98.52, 156.8)	124.14(98.12, 157.06)	8699.7	6.74(6.41)	106.67(87.82)
Orgasmic sensation decreased	74	228.89(176.43, 296.96)	228.64(177.21, 294.99)	12856.54	7.46(7.09)	175.5(141.15)
Psychogenic erectile dysfunction	8	600.71(237.07, 1522.11)	600.64(239.08, 1508.99)	2660.63	8.38(7.24)	334.13(153.48)
Catastrophic reaction	8	150.18(70.29, 320.85)	150.16(69.92, 322.5)	987.78	6.97(5.94)	125.3(66.39)
Psychophysiologic insomnia	5	288.79(102.95, 810.09)	288.77(102.19, 816.01)	1035.57	7.71(6.38)	208.83(88.1)
Thermophobia	3	150.17(43.47, 518.73)	150.16(43.68, 516.2)	370.42	6.97(5.41)	125.3(44.41)
**Congenital, familial and genetic disorders**						
Reproductive tract hypoplasia, male	5	234.64(85.96, 640.53)	234.63(86.35, 637.54)	886.21	7.48(6.18)	179(77.26)
Androgen insensitivity syndrome	4	375.42(113.04, 1246.79)	375.4(113.57, 1240.9)	995.74	7.97(6.48)	250.6(91.79)
Pseudohermaphroditism male	3	2252.5(234.29, 21655.66)	2252.4(236.45, 21455.83)	1687.8	9.14(7.24)	563.85(84.87)
**Surgical and medical procedures**						
Hair transplant	49	2832.01(1536.43, 5220.08)	2829.94(1541.35, 5195.83)	29054.83	9.21(8.68)	594.16(356.19)
Vasectomy	31	173.77(117.57, 256.84)	173.69(117.36, 257.05)	4322.65	7.14(6.6)	141.25(101.86)
Radical prostatectomy	15	154.31(88.52, 268.98)	154.27(89.11, 267.07)	1894.86	7(6.23)	128.15(80.5)
Varicocele repair	8	2002.37(531.2, 7547.99)	2002.13(528.04, 7591.37)	4363.93	9.09(7.85)	546.76(180.14)
Radiotherapy to prostate	8	333.73(145.1, 767.55)	333.69(146.5, 760.07)	1837.06	7.85(6.77)	231.32(115.23)
Scrotal operation	6	409.56(151.46, 1107.5)	409.53(150.72, 1112.77)	1582.17	8.05(6.79)	265.34(115.43)
Penile prosthesis insertion	6	128.72(54.14, 306.04)	128.71(54.33, 304.89)	649.04	6.78(5.63)	110.02(53.3)
Rectal fistula repair	3	173.27(49.37, 608.06)	173.26(49.42, 607.4)	417.46	7.14(5.57)	140.96(49.31)
Uvulopalatopharyngoplasty	3	125.14(36.86, 424.84)	125.13(37.12, 421.81)	316.65	6.75(5.21)	107.4(38.62)
**Nervous system disorders**						
Pudendal canal syndrome	12	214.55(112.95, 407.55)	214.51(112.34, 409.59)	1983.51	7.38(6.51)	167.07(97.66)

ROR, reporting odds ratio; CI, confidence interval; PRR, proportional reporting ratio; chisq, chi-squared; IC, information component; EBGM, empirical Bayesian geometric mean; IC025, the lower limit of 95% CI of the IC; EBGM05, the lower limit of 95% CI of EBGM; PTs: preferred terms

In order to improve the stability of the calculations with fewer cases [30], we also a1nalyzed the IC values and found that male pseudohermaphroditism male (n = 3, IC = 9.14), varicocele repair (n = 8, IC = 9.09), psychogenic erectile dysfunction (n = 8, IC = 8.38), epididymal tenderness (n = 3, IC = 8.14), scrotal operation (n = 6, IC = 8.05), and androgen insensitivity syndrome (n = 4, IC = 7.97) were among the unexpected signals with higher IC values. This implies a robust correlation with finasteride dosage.

To classify the early warning signals more carefully [31], we performed a descending order ranking of the BCPNN algorithm, and after excluding drug-unrelated signals, the top 10 finasteride PTs were post-5α reductase inhibitor syndrome (EBGM = 701.12), hair transplant (EBGM = 594.16), pseudohermaphroditism male (EBGM = 563.85), varicocele repair (EBGM = 546.76), male genital atrophy (EBGM = 449.35), organic erectile dysfunction (EBGM = 445.36), testicular failure (EBGM = 440.08), genital atrophy (EBGM = 418.62), epididymal cysts (EBGM = 387.77), and hypospermia (EBGM = 375.9).

In summary, we found that ejaculation disorder, sexual dysfunction, ejaculation disorder, and epididymal tenderness were consistent with the drug insert and dosing warnings. Unexpectedly post-5α reductase inhibitor syndrome, hair transplant, male pseudohermaphroditism male, Peyronie’s disease, male genital atrophy, epididymal cysts, testicular failure, varicocele repair, and androgen insensitivity syndrome were absent from the drug insert and require additional investigation.

### Time to onset analysis

**Fig 4:**
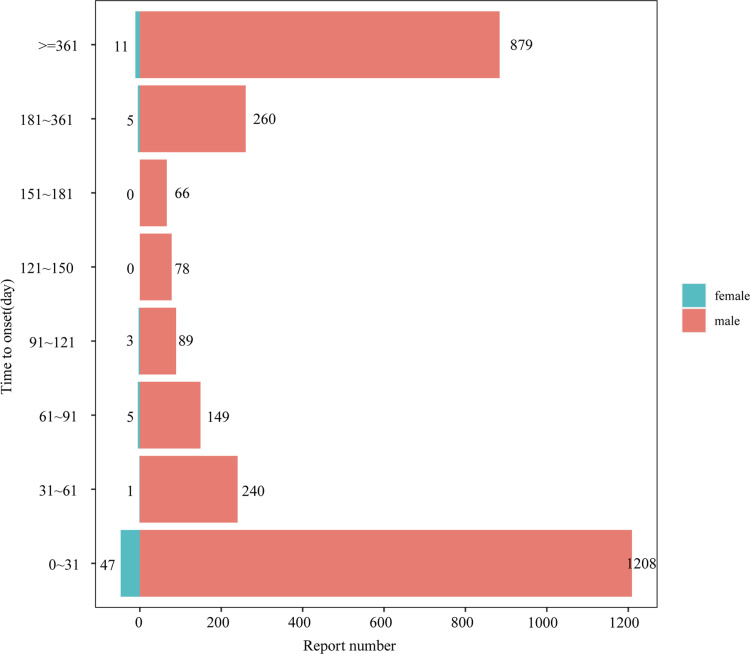
Time to onset of AEs in male and female patients receiving finasteride therapy. AEs: adverse events.

Out of all AEs reported, after excluding inaccurate, missing, or unknown sex time of onset reports, a total of 7019 included the onset time, with a median onset time of 61 days (interquartile range 6.00-463.75). In Fig 4, it was shown that the time of onset in males (n = 1208) versus females (n = 47) was predominantly within one month after initiation of finasteride medication. Interestingly, after one year of finasteride treatment, AEs could still occur in males (n = 879) and females (n = 11). This indicates the importance of ongoing patient monitoring for potential AEs even beyond one year of finasteride treatment.

### Subgroup analysis

#### Sex in different PT groups.

**Fig 5:**
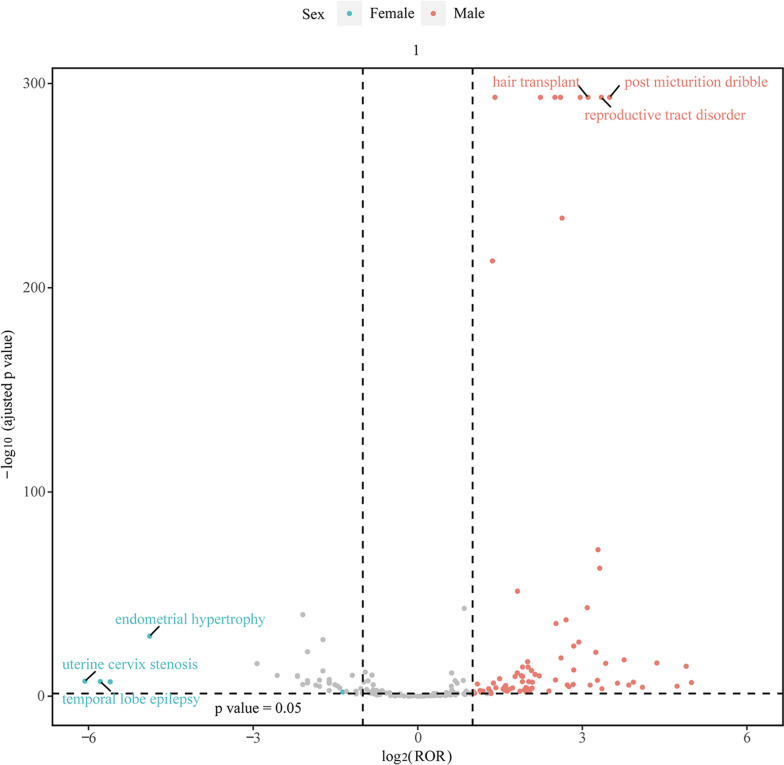
Sex-differentiated risk signal volcano plot for finasteride.

The study investigated the effect of sex on adverse effects of finasteride treatment by analysing 102 male and 7 female AEs using four statistical methods, with the results detailed in S4-S5 Tables. Subgroup analyses showed that depression and anxiety were common AEs in both sexes. The ‘volcano plot’ in Fig 5 visualised the sex-specific signals, with blue dots indicating female-associated AEs and red dots indicating male-associated AEs. Cervical stenosis was more common in women, while post-micturition dribbling was more common in men. These findings highlight sex differences in AEs associated with finasteride treatment and underline the need for sex-specific clinical management.

The horizontal coordinate shows the log2 PRR value and the vertical coordinate indicates the adjusted p-value after -log10 conversion PRR, proportional reporting ratio.

#### Age in different PT groups.

Age is a known risk factor for AGA [32], with the incidence of AGA increasing with age [33]. To minimise the confounding effects of age on the study of adverse reactions to finasteride, we performed an age-stratified analysis.

We evaluated 17 adverse reactions in three age groups: patients aged < 18 years, patients aged 18-65 years, and patients aged ≥ 65 years. The results, detailed in S6–S8 Tables, were arranged in descending order of the frequency of adverse reactions, and we excluded specific indications mentioned in the adverse reaction reports.In patients aged < 18 years, the three most common adverse reactions were depression (n = 11), erectile dysfunction (n = 10) and cryptorchidism (n = 8). In the 18-65 age group, the most common adverse reactions were erectile dysfunction (n = 1435), depression (n = 979) and anxiety (n = 704). In patients aged ≥ 65 years, the top three AEs were depression (n = 102), gynaecomastia (n = 73) and erectile dysfunction (n = 70). Common AEs across all age groups included erectile dysfunction, decreased libido, depression and suicidal ideation (S9 Table).

As most of the cases were less than 50 after subgroup analysis, we used IC values for descending order. The three most common AEs in patients aged < 18 years based on IC values were scrotal disorders (IC = 10.92), libido decreased (IC = 9.38) and loss of libido (IC = 9.01). In patients aged 18-65 years, the most common PTs were post-5-alpha reductase inhibitor syndrome (IC = 9.55), male genital atrophy (IC = 9.32), and genital atrophy (IC = 8.89). In patients aged ≥ 65 years, the most common PTs were prostate tenderness (IC = 9.98), penile shrinkage (IC = 9.57), and penile exfoliation (IC = 9.33). Finally, of all the PTs, the highest IC values in patients aged < 18 years were observed for reproductive system and breast disorders. For patients aged 18-65 years, the highest IC values were found for endocrine disorders. In patients aged ≥ 65 years, reproductive system and breast disorders again had the highest IC values. Common PTs in all age groups included erectile dysfunction, libido decreased, depression, suicidal ideation, psychotic disorders and disturbance in attention. These findings suggest that different age groups experience different AEs, with a different focus on SOCs.

## Discussion

To our knowledge, earlier studies on finasteride safety have primarily been confined to clinical trials or concentrated on particular AEs, such as sexual dysfunction, and have mainly investigated potential safety concerns [34–36], whereas our study discusses this more fully and extensively. Although in a large real-world drug study in 2017 it comprehensively and systematically summarized global reports of finasteride-associated sexual dysfunction in FAERS, we offered a more precise and comprehensive description and identification of AEs associated with finasteride. Although the incidence of AEs reported in clinical trials was low, the risk of occurrence of finasteride-associated AEs still exists, necessitating further studies and additions to the literature.

With regard to ‘reproductive system and breast disorders’, sexual dysfunction is the most significant adverse effect of finasteride treatment in young men with AGA, manifested by loss of libido and erectile dysfunction [37]. Moreover, the distribution of medication-related AEs indicated that the highest number of cases (3146 27.22%) were in the age group of 18-45 years, and in the younger age group even a low dose of finasteride (1mg) may lead to persistent sexual dysfunction in young males, which may increase the risk of suicide [38]and persist after discontinuation of the medication [8]. This may be due to the fact that younger patients have more stressful lives, are more sensitive to hormonal changes than older adults [39], are more prone to symptoms, and are more susceptible to the adverse effects of finasteride. These two adverse effects further confirm the existing knowledge about the potential adverse consequences of finasteride on sexual functioning and the increased risk of suicide [40]. It was found that an increased risk of suicide may exist with finasteride in the treatment of men with a history of psychological disorders [41]. Surprisingly, the 2023 Treatment Update for AGA shows that suicide and depression in patients with AGA may be caused by sexual dysfunction (erectile dysfunction, hypospermia, neurological dysfunction), a side effect of finasteride (1mg once daily) [42]. Therefore, comprehensive and precise clinical screening for the drug and close monitoring of patients for post-drug adverse effects are warranted.

With regard to the ‘psychiatric disorders’, psychological distress in patients with AGA has become a significant motivator in the development of effective treatments and the reduction of adverse effects of medication [43]. There is a risk of suicide, depression, and anxiety in patients younger than 40 years of age treated with finasteride for alopecia areata [44], which may be related to the involvement of 5-ARIs in the central nervous system’s synthesis of neuroactive steroidal organisms [45]. Neurosteroids are important physiological regulators of neural function in the adult brain and are involved in the control of the neuroendocrine system of reproduction [46]. A small human study observed depressive symptoms associated with reduced levels of neurosteroids in the cerebrospinal fluid of men who were taking finasteride [47]. This observation was supported by animal studies [48,49]. A study has been conducted by evaluating FMRI and HPLC-mass spectrometry analyses of neurosteroid levels in cerebrospinal fluid, pointing to the fact that finasteride disrupts neurotransmitters and chemical messengers [50]. These findings reinforce that anxiety and depression induced by finasteride use cannot be ignored. However, it has been suggested that the results of disproportionate analyses of AE signals may be influenced by the susceptibility of young patients to adverse factors, and further sensitivity analyses have shown an association between AGA and psychological AEs in young people that is not due to drug use [51]. This is consistent with the fact that pharmacovigilance does not directly prove biological causation. Emerging evidence also suggests that finasteride inhibits glioblastoma proliferation [52]. These findings highlight the potential relevance of finasteride to neuroscience and the need to closely monitor neurological psychiatric problems in patients receiving finasteride.

With regard to ‘renal and urinary disorders’, the only PT’s with positive signals were post micturition dribble and terminal dribbling, the former of which is the most frequent adverse reaction in men. Traish suggests that the symptoms are due to a novel tissue-specific androgen deficiency that develops when finasteride inhibits the enzyme 5-alpha reductase, which reduces the biosynthesis of 5alpha-dihydrotestosterone [53]. The symptoms of post micturition dribble have been shown to be associated with the development of a new form of androgen deficiency in men. In addition, finasteride has been widely used in the treatment of a variety of testosterone-related disorders, and new evidence suggests that finasteride has a protective effect on testosterone-induced calcium oxalate crystals, which is effective in preventing the formation of calcium oxalate-type renal stones and neutralising the calcium oxalate-promoting effects of testosterone [54]. Future studies could explore the relationship between finasteride and testosterone levels, and reduce the prevalence of renal stones in patients with high levels of the testosterone.

At the PT level, we discovered that while prostate pain, prostate calcification, and prostate atrophy were notable in disproportionate assessments and identified as adverse reactions by certain studies, they were regarded as treatment indications in Table 1, consistent with findings from another research. Although finasteride reduces the risk of prostate cancer, it increases the malignancy of high-grade prostate cancer [55]. To ensure the reliability of our results, we omitted adverse reactions listed as indications in Table 2.

In addition to the expected AEs associated with finasteride use, our study identified a number of unintended AEs not mentioned in the specification that need to be further analyzed and evaluated, including ‘Peyronie’s disease’, ‘post-5α reductase inhibitor syndrome’, ‘catastrophic reaction’ and “thermophobia”. Peyronie’s disease is a male condition characterized by penile curvature, pain, penile shortening and erectile dysfunction [56]. This is an unintended AE associated with AGA treated with finasteride (176/210 83.8%) [57]and the results of our study are consistent with the above studies. In addition, a case report found that the pathogenesis of finasteride-treated AGA is the same as that of Peyronie’s disease, in that inflammation affects the penile leukomalacia, leading to the formation of inelastic microtissue that deforms the penis [58,59]. However, the relationship needs to be further determined [57]. Another specification unmentionable AE that appeared in our analysis was the post 5-alpha-reductase inhibitor syndrome, 5-ARIs are drugs used to treat androgen-dependent diseases. Long-term use of 5-ARIs in patients with AGA leads to persistent side effects known as post-finasteride syndrome (PFS) [60,61]. PFS is also characterized by symptoms of penile shortening and penile curvature [62]. Not only this, but also persistent or irreversible sexual, neurological, physical and psychiatric side effects [63]. Leliefeld et al. have demonstrated that finasteride, a 5α-reductase inhibitor, inhibits the conversion of testosterone to 5α dihydrotestosterone (DHT) in the prostate and scalp and impairs the levels of other neurosteroids in the brain, demonstrating that sustained changes in androgen receptor and neurosteroid levels are associated with PFS [64]. However, previous studies have been inconclusive as to whether or not PFS exists [65]. Finally, our study reinforces the need for additional clinical research to assess the balance between the health risks and benefits of the medication. Careful consideration is required when using finasteride and developing potential new therapeutic options for treating these two conditions.

There is no mention of the adverse reaction in the insert: male breast cancer in the FAERS database or in the previous literature. As for the rare adverse reaction in the specification: hypersensitivity reaction, there was one case report of a patient with prostatic hyperplasia who developed maculopapular rash and rash on the extremities after 2 months of drug administration [66]. According to the FAERS database, SOCs not mentioned in the specification included endocrine system diseases, renal and urinary system diseases, various surgical and medical operations, and various congenital familial hereditary diseases. It should be noted that we conducted a revalidation of the results of the specification from a pharmacovigilance perspective for reproductive and breast disorders, psychiatric categories, and various neurological disorders. There is emerging evidence that finasteride both treats suppurative sweats in women [67] and reduces the incidence of bladder cancer [68,69]and induces type 2 diabetes [70], down-regulates androgen receptor expression in the renal cortex [71], forms blood clots [72], and alters intestinal microbial abundance [73,74] among other things. With regard to ‘endocrine disorders’ and ‘renal and urinary disorders’ it is noteworthy that almost all clinical trials did not include primary and secondary endpoint sensitisers for type 2 diabetes mellitus, renal dysfunction indicators. These unintended AEs raise concerns about the effects of finasteride on endocrine and renal disorders and the potential risk of severe endocrine disorders [75]. Further studies are necessary to investigate the prevalence of hormonal and metabolic changes and to evaluate the level of risk for individuals taking finasteride.

With respect to the timing of AEs and final outcome, the probability of AEs was greatest on days 0-31 of finasteride use in our analysis. Therefore, it is important to closely monitor potential risks associated with medication use over a one-month period. However, it is also possible that patients linked and reported their medication side effects within a short period of time after using the drug, which could also lead to bias in the data set. In addition, disability and life-threatening events such as male reproductive tract hypoplasia and catastrophic reactions were also reported in our study, suggesting that comprehensive monitoring and management of AEs receiving finasteride medication should not be underestimated, especially in the treatment of male patients with AGA and prostatic hyperplasia [76].

From the perspective of sex subgroups, male patients reported a higher incidence of AEs with finasteride compared to female patients. This sex difference can be understood from both sociological and biological perspectives. From the sociological point of view, firstly, the prevalence of AGA is on the rise globally, with 45.72% of men and 5.05% of women [77]. Furthermore, premenopausal women suffering from hair loss are mainly treated with minoxidil, oral contraceptives, and cosmetic products [78]. Finasteride, on the other hand, has been used to promote hair growth mainly after menopause [79,80]. As a result, the population of women who use finasteride is relatively small. Secondly, women prefer hair transplants and cosmetics to increase scalp hair fullness [81]. Finally, the safety of oral finasteride in patients with a history of gynaecological malignancy has not yet been clarified, which makes the use of this drug in the treatment of AGA more limited, especially when compared to male patients [82]. Biologically, AGA, previously thought to have an autosomal dominant mode of inheritance, has a reduced epistasis in females [83],this makes males more susceptible to AGA than females [84]. Furthermore, female follicle levels of type I and type II 5α-reductase and androgen receptors are half those of males. The percentage of females with a clinical diagnosis of AGA is then lower than that of males. In summary, we hypothesize that men experience more AEs than women because of the aforementioned biological and sociological factors, and further investigation into the underlying mechanisms and reasons is warranted.

At the age subgroup level, our study found that patients aged 18-65 years were more likely to report AEs involving finasteride than patients aged < 18 years and those patients aged ≥ 65 years. Because of the high correlation between AGA severity and age factors [85], AGA not only affects social functioning and emotional well-being in young men [86], but also affects sexual functioning and reduces quality of life in women aged 18-65 years [87]. Therefore, clinicians can kindly remind patients aged 18-65 years of the medication precautions when using it in the clinical setting, so that finasteride AEs will not go unnoticed in the non-medical setting.

## Limitation

It is crucial to recognize some constraints of our research, primarily arising from the fundamental features of pharmacovigilance databases. Firstly, up to 53.67% of the reports in our study originated from consumers (n = 6203), given the potential for inaccurate or misleading content in reports submitted by non-medical professionals due to limitations in their medical knowledge. Second, our data analyses failed to cover a wide range of unquantified confounding variables that may affect AEs, such as potential drug interactions, changes in treatment regimens, and results of laboratory tests and instrumental examinations. Additionally, the FAERS database is subject to incomplete or inaccurate data, particularly regarding drug therapy duration. Missing information on treatment cessation and adherence may impact the timing of AEs. Finally, further prospective studies are needed to confirm and clarify the relationship between finasteride and these AEs, addressing issues like data inconsistency and confounding factors.

## Conclusions

We performed pharmacovigilance analyses using actual data from the FAERS database, and the adverse reactions identified in the survey were generally consistent with those in the package inserts. Additionally, we detected AEs not listed in the specifications, including post 5-alpha-reductase inhibitor syndrome, Peyronie’s disease, testicular microlithiasis, and catastrophic reactions. The identification of these strongly indicating AEs supplements the inherent limitation of the relatively small sample size in clinical studies of this drug. The results of this study help to inform the safe and rational use of the drug in the clinic.

## Supporting information

S1 Table Four grid table.
(XLSX)

S2 Table Four major algorithms used for signal detection.
(XLSX)

S3 Table Signal strength of reports of finasteride administration at the SOC level
(XLSX)

S4 Table Signal strength of reports of finasteride administration in female at the PT level.
(XLSX)

S5 Table Signal strength of reports of finasteride administration in male at the PT level.
(XLSX)

S6 Table Signal strength of reports of finasteride administration at the PT level (age < 18).
(XLSX)

S7 Table Signal strength of reports of finasteride administration at the PT level (age = 18-65).
(XLSX)

S8 Table Signal strength of reports of finasteride administration at the PT level (age>=65).
(XLSX)

S9 Table Number of adverse reaction case reports of finasteride administration at the PT level.
(XLSX)
