# Supplementary material for: Multidimensional assessment of adverse events of finasteride：a real-world pharmacovigilance analysis based on FDA Adverse Event Reporting System (FAERS) from 2004 to April 2024
Source: PLoS One. 2025 Mar 24;20(3):e0309849. doi: 10.1371/journal.pone.0309849 (PMC11932486; doi:10.1371/journal.pone.0309849)
Supplement: Attachment — Submitted filename: PLOSOne Human Subjects Research Checklist.docx [file pone.0309849.s010.docx]

**Human Participants Research Checklist**

***Complete the following if your study involved human participants or human participants’ data. These questions should be addressed for prospective and retrospective studies.***

1. Did you obtain ethics approval for this study?
   - If yes, please upload (file type “Other”) the original approval document you received from your ethics committee. If the original document is in another language, please also provide an English translation.

___ Uploaded	_√__ N/A

- If you did not obtain ethical approval, please explain why this was not required below.

Ethical review and approval were not required for the study in human participants in accordance with local legislation and institutional requirements. Written informed consent from [patient/participant OR patient/participant's legal guardian/next of kin] was not required for participation in this study in accordance with national laws and institutional requirements.

This study aims to evaluate the adverse events (AEs) associated with finasteride use, based on data from the US Food and Drug Administration Adverse Event Reporting System (FAERS).

The U.S. FDA Adverse Event Reporting System (FAERS) database is a freely accessible database created by the FDA to collect cases of adverse drug reactions (ADRs) from around the world[1,2].

Referring to the type of article in your magazine：

https://doi.org/10.1371/journal.pone.0298609;

https://doi.org/10.1371/journal.pone.0281983

References

[1] Nagai, J., and Ishikawa, Y. (2021). Analysis of anticholinergic adverse effects using two large databases: the US food and drug administration adverse event reporting system database and the Japanese adverse drug event report database. PLoS One 16, e0260980. doi:10.1371/journal.pone.0260980

[2] Li D, Chai S, Wang H, Dong J, Qin C, Du D, Wang Y, Du Q, Liu S. Drug-induced QT prolongation and torsade de pointes: a real-world pharmacovigilance study using the FDA Adverse Event Reporting System database. Front Pharmacol. 2023 Dec 21;14:1259611. doi: 10.3389/fphar.2023.1259611.

2. If you prospectively recruited human participants for the study – for example, you conducted a clinical trial, distributed questionnaires, or obtained tissues, data or samples for the purposes of this study, please report in the Methods:
   i. the day, month and year of the **start and end** of the recruitment period for this study.
   ii. whether participants provided informed consent, and if so, what type was obtained (for instance, written or verbal, and if verbal, how it was documented and witnessed). If your study included minors, state whether you obtained consent from parents or guardians. If the need for consent was waived by the ethics committee, please include this information.

___ Completed	_√__ N/A

3. If you are reporting a retrospective study of medical records or archived samples, please report in the Methods section:
i. the day, month and year when the data were accessed for research purposes
ii. whether authors had access to information that could identify individual participants during or after data collection

___ Completed	_√__ N/A
