# Supplementary material for: Multidimensional assessment of adverse events of finasteride：a real-world pharmacovigilance analysis based on FDA Adverse Event Reporting System (FAERS) from 2004 to April 2024
Source: PLoS One. 2025 Mar 24;20(3):e0309849. doi: 10.1371/journal.pone.0309849 (PMC11932486; doi:10.1371/journal.pone.0309849)
Supplement: Attachment — Submitted filename: Response to Reviewers.docx [file pone.0309849.s011.docx]

**Journals' comments:**

**Comment 1: Please ensure that your manuscript meets PLOS ONE's style requirements, including those for file naming.**

**Response 1:** Thank you for your suggestion. I have carefully reviewed the manuscript and ensured that it complies with PLOS ONE's style requirements, including those related to file naming.

**Comment 2: We note that Figure 2 in your submission contain [map/satellite] images which may be copyrighted.**

**Response 2:** Thank you for your valuable feedback. According to the journal's map copyright guidelines, "If you created the map in a software program like R or ArcGIS, please locate the source of the basemap within the package used to generate the map." Figure 2 was created using the mapdata package in R software, adding the basemap source (<https://cran.r-project.org/web/packages/mapdata/index.html>) to the manuscript.

**Page 12, Lines 309-311.**

**Comment 3: Please include your tables as part of your main manuscript and remove the individual files. Please note that supplementary tables (should remain/ be uploaded) as separate "supporting information" files.**

**Response 3:** Thank you for your suggestion. We have incorporated Table 1 and Table 2 into the main manuscript and removed the individual files. The supplementary tables have been uploaded as separate "supporting information" files as requested.

**Comment 4: Please review your reference list to ensure that it is complete and correct. If you have cited papers that have been retracted, please include the rationale for doing so in the manuscript text, or remove these references and replace them with relevant current references. Any changes to the reference list should be mentioned in the rebuttal letter that accompanies your revised manuscript. If you need to cite a retracted article, indicate the article’s retracted status in the References list and also include a citation and full reference for the retraction notice.**

**Response 4**: Thank you for your thorough feedback. We have carefully reviewed the reference list to ensure it is complete and accurate. Each reference has been checked individually, and we confirm that no retracted papers have been cited.

**Comment 5: While revising your submission, please upload your figure files to the Preflight Analysis and Conversion Engine (PACE) digital diagnostic tool,** [**https://pacev2.apexcovantage.com/**](https://pacev2.apexcovantage.com/)**.**

Response 5：Thank you for your suggestion, the chart file has been uploaded to the Pre-Check Analysis and Conversion Engine (PACE) digital diagnostic tool for proofreading.

**Reviewers' comments:**

**Reviewer #1:**

**Comment 1: The authors use four statistical tools to detect the suspected AEs of finasteride use using the real-life patient reports. In their analysis, an AE signal that meets the criteria for any of these four methods is considered as a drug-associated AE. However, these algorithms have multiple strengths and weaknesses over each other, and AEs that have highest overlap of signals would be more reliable to identify as finasteride associated. To reflect the overlap and differences between these methods, a heatmap of the signals of AEs detected by ROR, PRR, BCPNN, and MGPS methods at the SOC level will be useful to include.**

**Response 1:** Thank you for your valuable suggestion. In response, we have added **Figure 3**, which presents a heatmap of AE signals detected by the ROR, PRR, BCPNN, and MGPS methods at the SOC level. This provides a more intuitive visualization of the data presented in S3_Table.

**Page 13, Line 328.**

**Comment 2: The authors state their suggestions for finasteride use and further investigation of the biology of this drug based on their analysis of patient AE reports. Although, these are insightful findings and recommendations, it is important to mention the biases in these datasets. For instance, in the Discussion section, the authors suggest close monitoring for potential risks of this drug over a month based on the high number of reports in this time period. However, patients will tend to link and report side effects that they experience in a short time after they start to use the drug, resulting in a biased dataset.**

**Response 2:** Thank you for your insightful comment. In response, we have addressed this concern in the Discussion section by adding the statement: “However, it is also possible that patients linked and reported their medication side effects within a short period of time after using the drug, which could also lead to bias in the data set.”

**Page 28, Lines 707–709.**

**Comment 3: Since the study focuses on the biological differences of patients using “sex” instead of “gender” is more appropriate. The authors use these words interchangeably through the text.**

**Response 3:** Thank you for your valuable suggestion. We have carefully revised the manuscript to use **“sex”** instead of **“gender”** throughout the text to ensure appropriate terminology is used.

**Comment 4: There are repetitive and incomplete sentences that must be fixed.
Examples include:
- “These methods were used to evaluate the of finasteride medication and AEs. whether there is a significant association between finasteride drug use and AEs.”
- “Finally, by means of the Medex UIMA 1.8.3 system the standardization of drug
names[21,22]. Finally, by means of the Medex UIMA 1.8.3 system the standardization of drug names.”
- “This is probably due to the fact that the younger patients have a more stressful life and are more sensitive to hormonal changes than the older ones[39]. In addition, in the PT analysis we observed ‘sexual dysfunction’ and ‘catastrophic reaction’. This may be due to the fact that younger patients have more stressful lives, are more sensitive to hormonal changes than older adults, are more prone to symptoms, and are more susceptible to the adverse effects of finasteride.”**

**Response 4:**

Thank you for your valuable feedback. We have addressed the repetitive and incomplete sentences and checked that there are no similar errors elsewhere in the full manuscript, as follows:

- The repetitive phrase “the of finasteride medication and AEs” has been deleted.
  **Page 2, Line 41.**
- The redundant sentence “Finally, by means of the Medex UIMA 1.8.3 system the standardization of drug names” has been removed.
  **Page 6, Line 181.**
- The duplicated sentences “This is probably due to the fact that the younger patients have a more stressful life and are more sensitive to hormonal changes than the older ones. In addition, in the PT analysis we observed ‘sexual dysfunction’ and ‘catastrophic reaction’. This may be due to the fact that younger patients have more stressful lives, are more sensitive to hormonal changes than older adults” have been removed.
  **Page 23, Line 561**
- As per your suggestion, we have made revisions to other repetitive sections of the manuscript. Specifically, the sentence "After excluding inaccurate, missing, or unknown gender time of onset reports, a total of 7019 finasteride-administered AEs reported time of onset." has been removed.

**Page 20, Lines 420.**

**Comment 5: Abbreviations should be spelled out in the text where they are used the first time such as Benign prostatic hyperplasia (BPH).**

**Response 5:** Thank you for your valuable suggestion. We have revised the manuscript to ensure that abbreviations are spelled out in full the first time they appear in the text, such as “Benign prostatic hyperplasia (BPH)”.

**Page 4, Lines 110–111.**

**Comment 6: Word missing: “These methods were used to evaluate the XXX of finasteride medication and AEs.”**

**Response 6:** Thank you for your helpful feedback. We have corrected the expression by removing the phrase “the of finasteride medication and AEs”.

**Page 2, Line 41.**

**Comment 7: Space missing: “Emergingevidence”**

**Response7：**Thank you for your careful observation. We have corrected the phrase “Emergingevidence” to “Emerging evidence”.

**Page 25, Line 625.**

**Comment 8: PT should be capitalized: “… the only pt’s with positive signals …”**

**Response 8:** Thank you for your valuable feedback. We have made the necessary correction and changed “pt’s” to “PT’s”.

**Page 25, Line 627.**

**Reviewer #2:**

**Comment 1: Adverse effects should be summarized in tabulated form alongwith their relation to demographics & external factors**

**Response 1:** Thank you for your insightful suggestion. In response, we have summarized the adverse effects in relation to demographics and external factors. The gender differences are presented in Figure 5 as a heatmap, and the relationship between adverse effects and age subgroups has been added to **S9_Table**.

We fully agree with the reviewers on the importance of considering external factors, such as environmental or genetic influences, when analyzing adverse effects. However, as the database used for this study only provides limited demographic data (including year, sex, age, reporter, reported countries, route, outcomes, time to onset, and indications, as shown in Table 1), we acknowledge that our analysis of these external factors is constrained. Despite these limitations, we have endeavored to extract as much valuable information as possible from the available data, ensuring a thorough analysis aligned with the study's primary objectives.

**Page 22, Line 511**
**Page 45, Line 1116-1117**

**Comment 2: Duration of treatment with demographics should be statistically analyzed**

**Response 2:** Thank you for your valuable suggestion. While we understand the importance of analyzing the duration of treatment along with demographics, we must note that the FAERS data used in this study is primarily focused on drug safety signal analysis. Unfortunately, the data regarding treatment duration is often incomplete or unreliable, as some reports lack specific dates, particularly the end date of treatment. Given these limitations, we feel that conducting a detailed statistical analysis of treatment duration in this context may not provide accurate insights. We believe that further investigation into the role of treatment duration would be better suited for clinical trial data.We explain this in the **Limitation** section.

We appreciate your understanding and thoughtful feedback.

**Page 29, Line 769-773**

**Comment 3:** Doses of the drug should be mentioned at which ADRs appeared

**Response 3:** Thank you for your helpful suggestion. We have added the drug dose at which the ADRs appeared, specifying “1mg once daily”, and have included the relevant reference [1].

**Page 24, Line 597-599**
**Page 37, Lines 951-952**

**Comment 4: How anxiety & depression was analyzed? Which scale was used?**

**Response 4:** Thank you for your enlightening question. Unfortunately, the FAERS database (https://open.fda.gov/data/downloads/) does not include specific scales for anxiety and depression, so this information is not available. In addition, the primary focus of this study is on the adverse events (AEs) reported or not reported in the finasteride drug label and the relationship between AEs and age and sex.

As a comprehensive sensitivity analysis was conducted in a 2021 JAMA study that found no causal association between androgenetic alopecia and psychological adverse events in younger individuals, we did not pursue an in-depth analysis of this issue. We have added this clarification in the Discussion section and included the relevant reference [2].

**Page 24, lines 615-627**

**Page 39, lines 983-985**

**Comment 5: Make the findings more summarized**

**Response 5:** Thank you for your helpful suggestion. In response, we have revised the Results section to make the findings more concise and summarized. The relevant sections have been updated as follows:

**Page 2, Lines 46–62**

**Page 9, Lines 254–271**
**Page 13, Lines 316–339**
**Page 13, Lines 341–346**
**Page 21, Lines 454–461**
**Page 22, Lines 466–542**

[1] Devjani S, Ezemma O, Kelley KJ, Stratton E, Senna M. Androgenetic Alopecia: Therapy Update. Drugs. 2023;83: 701–715. doi:10.1007/s40265-023-01880-x

[2] Choi JW, Huh CH, Choi GS. Association of Hair Loss With Suicidality and Psychological Adverse Events vs Finasteride Use. JAMA Dermatol. 2021;157: 737. doi:10.1001/jamadermatol.2021.1001
