# Supplementary material for: Multidimensional assessment of adverse events of finasteride：a real-world pharmacovigilance analysis based on FDA Adverse Event Reporting System (FAERS) from 2004 to April 2024
Source: PLoS One. 2025 Mar 24;20(3):e0309849. doi: 10.1371/journal.pone.0309849 (PMC11932486; doi:10.1371/journal.pone.0309849)
Supplement: Attachment — Submitted filename: pone.0309849.docx [file pone.0309849.s012.docx]

**Multidimensional assessment of adverse events of finasteride：a real-world pharmacovigilance analysis based on FDA Adverse Event Reporting System (FAERS) from 2004 to April 2024**

Xiaoling Zhong^2¶^,Yihan Yang^3¶^,Sheng Wei^4^,Yuchen Liu^1*^

^1^ Department of Acupuncture and Moxibustion, Shenzhen Luohu District Hospital of Chinese Medicine, Shenzhen, China

^2^ Department of Acupuncture, Guangdong Provincial Hospital of Traditional Chinese Medicine, Guangzhou, China

^3^ The Institution of Rehabilitation Industry, Fujian University of Traditional Chinese Medicine, Fuzhou, China

^4^ Department of General Medicine, The Second Affiliated Hospital of Wannan Medical College, Wuhu, China

^*^Corresponding author

Email:1109996928@qq.com(YL)

^¶^These authors contributed equally to this work.

# Abstract

## Background

Finasteride is commonly utilized in clinical practice for treating androgenetic alopecia, but real-world data regarding the long-term safety of its adverse events(AEs) remains incomplete, necessitating ongoing supplementation. This study aims to evaluate the AEs associated with finasteride use, based on data from the US Food and Drug Administration Adverse Event Reporting System (FAERS), to contribute to its safety assessment.

## Methods

We reviewed AE reports associated with finasteride from the US Food and Drug Administration Adverse Event Reporting System database, covering the period from the first quarter of 2004 to the first quarter of 2024. We assessed the safety of finasteride medication and AEs using four proportional disproportionality analyses: reported odds ratio (ROR), proportionate reporting ratio (PRR), Bayesian Confidence Propagation Neural Network (BCPN), and Multi-Item Gamma Poisson Shrinkage (MGPS). These methods were used to evaluate whether there is a significant association between finasteride drug use and AEs. To investigate potential safety issues related to drug use, we further analyzed the similarities and differences in the onset time and AEs by sex, as well as the similarities and differences in AEs by age.

## Results

A total of 11,557 AE reports in which finasteride was the primary suspected drug were analysed. The majority of patients were male (86.04%) and a significant proportion were young adults aged 18-45 years (27.22%). A total of 73 different AEs were categorised into 7 system organ classes (SOCs), with common AEs including erectile dysfunction and sexual dysfunction. In addition, we identified previously unlisted AEs, including Peyronie's disease and post-5α reductase inhibitor syndrome. Of the reported AEs, 102 occurred in men and 7 in women, with depression and anxiety being significant AEs observed in both sexes. When analysed by age group, there were 17 AEs in patients aged <18 years, 157 in patients aged 18-65 years and 133 in patients aged ≥65 years. Common AEs in all age groups included erectile dysfunction, decreased libido, depression, suicidal ideation, psychotic disturbances and attention disorders. The median time to onset of all AEs was 61 days, with the majority occurring within the first month of treatment. Notably, a significant number of AEs persisted beyond one year of treatment.

## Conclusion

The results of our study uncovered both known and novel AEs associated with finasteride medication. Some of these AEs were identical to the specification, and some of them signaled AEs that were not demonstrated in the specification. In addition, some AEs showed variations based on sex and age in our study. Consequently, our findings offer valuable insights for future research on the safety of finasteride medication and are anticipated to enhance its safe use in clinical practice.

**Keywords: real-world pharmacovigilance analysis, FAERS, pharmacovigilance, finasteride**

# Introduction

Androgenetic alopecia (AGA), commonly referred to as male-pattern baldness, is characterized by hair loss driven by dihydrotestosterone, a potent derivative of testosterone[1].It has a significant negative physiological and psychological impact on patients. Patients with AGA experience adverse effects such as sexual dysfunction, affective disorders, stigma and low self-esteem compared to normal individuals[2,3], especially in younger patients [4]. Fortunately, however, since receiving approval for treating AGA in 1997, finasteride has gained popularity for AGA treatment and has shown positive therapeutic outcomes[5].

Finasteride, as the main drug for the treatment of AGA, works by efficiently inhibiting 5-alpha reductase inhibitors (5-ARIs)[6]. Since the synthesis of sex hormones in the body is dependent on steroid reductases, and SRD5A2 is a biofilm chimeric steroid in the class of steroid reductase enzymes, SRD5A2 can synthesize dihydrotestosterone by catalyzing the reductive reaction of testosterone, which can lead to the onset of AGA if it is too high[7]. So finasteride as 5-alpha reductase inhibitor is the anti-androgenic drug that is used to treat both diseases and is widely available[8]. It is noteworthy that patients with Benign prostatic hyperplasia and AGA experience AEs of depression or suicide when neuroactive steroids have a reduced ability to control mood, behavior, and cognition[9–11]. The data show that between 1998 and 2008, an estimated 4.6 million men were treated with at least 1 mg of finasteride[12]. More importantly, as of June 2021, the FDA AE disclosure data have reported up to 10,295 serious AEs with finasteride use. Unfortunately, however, safety information regarding finasteride in patients with AGA primarily stems from clinical trials and post-marketing observational studies. The frequency of persistent AEs associated with it remains unclear, underscoring the necessity for additional evidence-based research to investigate the potential risks of finasteride use[13]. A Real-world study, with their large data samples and rigorous inclusion and exclusion criteria, have emerged as a useful tool to explore AEs and to compensate for clinical trials. An effective way of exploring adverse drug events and bridging the gap between clinical trials to provide us with evidence-based information.

FAERS is an openly available database created by the FDA that gathers instances of adverse drug events reported globally, with a large amount of real-world data and extensive geographic coverage of records of AEs from practicing physicians, pharmacists, registered nurses, consumers, and other healthcare professionals, among others[14,15]. Being the largest pharmacovigilance database globally, FAERS is an invaluable resource for identifying AEs associated with drug usage[16]. Thus, the objective of this study was to assess the long-term safety profile of finasteride following its market introduction using data extracted from FAERS database[17]. This study offers guidance and citations for the rational and safe clinical administration of finasteride dosage.

# Materials and methods

## Date sources

FAERS is a database for public post-marketing safety monitoring of drugs, and this retrospective analysis of adverse drug reactions utilized data sourced from the FAERS database. Our study gathered data from the FAERS Quarterly Data Extract Files spanning from Q1 2004 (FDA approval of finasteride) through Q1 2024.The FAERS database is updated quarterly and is widely recognized internationally for its large volume of data and standardization, and all the data within the FAERS database can be freely downloaded (<https://open.fda.gov/data/downloads/>).

## Data extraction

The FAERS data file contains seven comprehensive datasets: drug information (DRUG), information on adverse events (REAC), information on patient demographics and management (DEMO), information on patient outcomes (OUTC), information on the source of the report (RPSR), date of initiation and termination of medication (THER), and information on the indication of the drug (INDI). Whereas, the role of the reporting drug in each case was indicated in the DRUG table through specific codes: PS (primary suspect drug), SS (secondary suspect drug), C (concomitant drug), and I (interacting drug)[18]. Major endpoints were identified as disability (DS), hospitalisation (HO), life-threatening (LT), death (DE), and need for intervention to prevent permanent injury/damage (RI), congenital anomalies (CA), and other serious medical events (OT). In this study, cases were identified by utilizing drug names listed in the DRUG file, and selections were determined based on PS outcomes.

AEs in the FAERS database were categorized using the international medical dictionary MedDRA, which organizes terms into a structural hierarchy comprising preferred term (PT), system organ class (SOC), high level group term (HLGT), high level term (HLT), and lowest level term (LLT).We meticulously identified individual AE) reports linked to finasteride at the SOC and PT levels. Additionally, our study concentrated on analyzing the specific drugs documented in the drug files, namely finasteride (generic name) and proscar (trade name), with the aim of obtaining a standardization of the AEs associated with finasteride after a name change[19,20]. Finally, by means of the Medex UIMA 1.8.3 system the standardization of drug names[21,22].

## Data cleaning

A retrospective pharmacovigilance investigation was undertaken to assess the post-market safety of finasteride. Data were sourced from the openly accessible FAERS database, encompassing raw FAERS data related to finasteride spanning from the first quarter of 2004 to the first quarter of 2024. All data retrieved from the US FDA website were imported into R (Version 4.2.2) for subsequent analysis. Duplicate reports were identified and eliminated in accordance with FDA guidance, applying this rigorous methodology aimed at avoiding duplicate entry of AE reports and ensuring further analysis following a thorough evaluation of the safety profile of finasteride[23]. The operation was performed in the following manner, selecting the PRIMARYID, CASEID, and FDA_DT fields of the DEMO table and sorting the data by CASEID, FDA_DT, and PRIMARYID; when reports with the same identical CASEID are available, the report with the largest FDA_DT value is retained; when records with the same CASEID and FDA_DT are available, the record with the largest PRIMARYID value is retained[24]. Not only that, but the erroneous case reports noted on the FDA website were deleted as suggested, and the final obtained clinical characteristics of the patients who were administered finasteride were obtained Clinical characteristics including sex, age, reporting region, reporter, time of reporting, and outcome were the relevant AE data results.

## Statistical analysis

The four-grid proportional imbalance method, known for its widespread application in pharmacovigilance studies, was employed for the disproportionality analysis investigating potential associations between finasteride and all reported AEs[25], as documented in S1 Table. Reported dominance ratio (ROR), proportional reporting ratio (PRR), information component (IC) in Bayesian confidence propagation neural network (BCPNN), and multinomial gamma poisson shrinker (MGPS) are the four main specific metrics computed using standard formulas for evaluating potential correlations involving finasteride and AEs[26].

In our study, the signals of the 4 AE recordings of the target drug were calculated, and a positive drug-associated AE signal was considered if it met at least one of the four algorithms (lower limit of 95% CI > 1, N ≥ 3; PRR ≥ 2, χ2 ≥ 4, N ≥ 3; IC025 > 0 or EBGM05 > 2) [27,28]. The methodologies and thresholds for the AE signals are detailed in S2 Table, while the procedural flowchart is depicted in Fig 1.

**Fig 1. Flowchart depicting the selection of the study population.**

## The flow diagram of selecting finasteride therapy related AEs from the FAERS database. DEMO, demographic and administrative information; FAERS, US Food and Drug Administration Adverse Event Reporting System.

## Time to onset (TTO) analysis

The onset time of AEs and the likelihood of a severe outcome were computed, with time to onset defined as the interval between EVENT_DT (date of AE onset) and START_DT (time of medication initiation), and median and interquartile ranges were used to describe the time to onset. In addition, AEs attributable to the route of administration were counted, calculated as number of serious outcomes/total number of events reported.

## Visualization of Data

Images were produced using the ggplot2 package and GraphPad Prism 8.0.1. We employed a global heat map to depict data from countries that submitted reports (https://cran.r-project.org/web/packages/mapdata/index.html). A line graph was utilized to illustrate the trend in case numbers from the first quarter of 2004 to the first quarter of 2024. Additionally, to ascertain if the AE signal differed between males and females following finasteride administration, we created a volcano plot displaying log2-transformed PRP values on the x-axis and -log10-transformed adjusted P-values on the y-axis[24]. When PRP exceeded 1 and P.adj was greater than 0.05, it suggested a distinct AE signal between female and male patients. The demographic ratios of reported cases by sex and age, along with annual case counts, were processed and visualized using Excel tables.

## Ethics statement

The study involving human participants did not require ethical review and approval in compliance with local laws and institutional guidelines. Written informed consent from participants or their legally authorized representatives was not necessary for participation in this study in accordance with national regulations and institutional policies.

# Results

## Characteristics of reported case

From the first quarter of 2004 to the first quarter of 2024, a total of 20,689,674 adverse reaction reports were submitted, of which 11,557 were specifically related to finasteride use. Table 1 presents comprehensive details of the reports, encompassing patient demographics such as sex and age, the reporting year, the occupation of the reporter, and the country from which the report was submitted. The peak number of finasteride-related reports occurred in 2024 (1,141 cases, 9.87%), followed by 2015 (1,000 cases, 8.65%) (Fig 2A). Geographical information was not available for 27.44% of the reports, limiting insight into regional trends (Fig 2B). Reports were predominantly from the United States (46.68%), United Kingdom (16.19%), Italy (3.51%), France (3.35%) and Germany (2.83%). The majority of reports involved male patients (86.04%), 3.44% were female and 10.52% were of unknown sex (Fig 2C). The majority of reports were submitted by consumers (53.67%) and physicians (20.73%) (Fig 2D). In terms of age, the largest group of patients were aged 18-45 years (27.22%), followed by those aged 45-65 years (9.44%), and 7.81% were ≥75 years, with 48.80% of the age information being unknown (Fig 2E). The most commonly reported indications for finasteride use were alopecia (28.36%), androgenetic alopecia (16.68%) and benign prostatic hyperplasia (9.06%) (Fig 2F). Final patient outcomes included disability in 22.60% of cases and hospitalisation in 14.55% of cases (Fig 2G).

**Table 1. Clinical characteristics of reports with finasteride as the primary suspected drug from the FAERS database.**

| **Variable** | **Total（%）** |
| --- | --- |
| **Year** |  |
| 2004 | 118(1.02) |
| 2005 | 131(1.13) |
| 2006 | 150(1.30) |
| 2007 | 153(1.32) |
| 2008 | 147(1.27) |
| 2009 | 172(1.49) |
| 2010 | 216(1.87) |
| 2011 | 399(3.45) |
| 2012 | 624(5.40) |
| 2013 | 652(5.64) |
| 2014 | 978(8.46) |
| 2015 | 1000(8.65) |
| 2016 | 833(7.21) |
| 2017 | 559(4.84) |
| 2018 | 641(5.55) |
| 2019 | 704(6.09) |
| 2020 | 975(8.44) |
| 2021 | 707(6.12) |
| 2022 | 581(5.03) |
| 2023 | 676(5.85) |
| 2024 | 1141(9.87) |
| **Sex** |  |
| female | 397( 3.44) |
| male | 9944(86.04) |
| unknown | 1216(10.52) |
| **Age_yr** | 41.00(29.00,67.00) |
| **Age_yrQ** |  |
| 0~18 | 66( 0.57) |
| 18~45 | 3146(27.22) |
| 45~65 | 1091( 9.44) |
| 65~75 | 711( 6.15) |
| >=75 | 903( 7.81) |
| unknow | 5639(48.80) |
| **Reporter** |  |
| Consumer | 6203(53.67) |
| Physician | 2396(20.73) |
| Other health-professional | 1098( 9.50) |
| Pharmacist | 975( 8.44) |
| unknown | 556( 4.81) |
| Lawyer | 327( 2.83) |
| Registered Nurse | 2( 0.02) |
| **Reported countries** |  |
| United States | 5395(46.68) |
| other | 3171(27.44) |
| United Kingdom | 1871(16.19) |
| Italy | 406( 3.51) |
| France | 387( 3.35) |
| Germany | 327( 2.83) |
| **Route** |  |
| oral | 7513(65.01) |
| other | 3956(34.23) |
| transplacental | 59( 0.51) |
| buccal | 15( 0.13) |
| topical | 14( 0.12) |
| **Outcomes** |  |
| other serious | 5476(52.48) |
| disability | 2358(22.60) |
| hospitalization | 1518(14.55) |
| life threatening | 500( 4.79) |
| death | 282( 2.70) |
| required intervention to Prevent Permanent Impairment/Damage | 193( 1.85) |
| congenital anomaly | 108( 1.03) |
| **Time to onset** | 61.00(6.00,463.75) |
| **Time to onsetQ** |  |
| 0~31 | 1337(19.05) |
| 31~61 | 260( 3.70) |
| 61~91 | 163( 2.32) |
| 91~121 | 102( 1.45) |
| 121~150 | 80( 1.14) |
| 151~181 | 71( 1.01) |
| 181~361 | 278( 3.96) |
| >=361 | 923(13.15) |
| unknow | 3805(54.21) |
| **Indications** |  |
| alopecia | 3297(28.36) |
| androgenetic alopecia | 1939(16.68) |
| benign prostatic hyperplasia | 1053( 9.06) |
| male pattern baldness | 128( 1.10) |
| others | 784( 6.74) |
| product used for unknown indication | 2117(18.21) |
| prostatic disorder | 103( 0.89) |
| prostatomegaly | 487( 4.19) |
| unknown | 1716(14.76) |

**Fig 2. Basic information and patient characteristics according to the report.**

(A) The annual distribution of finasteride administration related AEs reports from 2004 to April 2024. (B) Country distribution of AEs for finasteride administration, brighter colors represent a higher number of reports, Map created in R using mapdata package (<https://cran.r-project.org/web/packages/mapdata/index.html>) and the FAERS database (<https://open.fda.gov/data/downloads/>). (C) Sex ratio of male and female in reported events. (D) Occupational information ratio in reported events. (E) Age distribution ratio in reported events. (F) Ratio of indications in reported events. (G) Ratio of outcomes in reported events. Visualization through proportional area map, larger areas represent more reporters.

## Signals detection based on SOC levels

Statistical analysis of finasteride dose-related SOCs showed that 24 were affected (S3 Table, Fig 3). Among these, reproductive and breast disorders, endocrine disorders, and psychiatric disorders showed the highest associations based on PRR ≥2, χ2 ≥4, and N ≥3. Specifically, reproductive and breast disorders had the strongest association (ROR=26.68, PRR=21.97, χ2=241136.36, IC=4.42, EBGM=21. 37), followed by endocrine disorders (ROR=6.58, PRR=6.49, χ2=5179.48, IC=2.69, EBGM=6.44) and psychiatric disorders (ROR=4.57, PRR=3.77, χ2=32212.4, IC=1.91, EBGM=3.76). When ranked by number of cases, the most commonly reported disorders were psychiatric (n=14951), reproductive and breast (n=12293), neurological (n=5992), renal and urological (n=1841), surgical and medical manipulation (n=1268), endocrine (n=1122), and various congenital familial genetic disorders (n=282). These findings highlight key areas where finasteride-related AEs are commonly observed.

**Fig 3. Signal strength of reports of finasteride administration at the SOC level.**

SOC distribution of adverse reactions associated with finasteride administration, with darker colours representing stronger signals.

Analysis of adverse reactions showed a strong correspondence with the SOCs listed in the drug insert, confirming the reliability of the data. The SOCs for which adverse reactions appeared in the drug insert included skin and subcutaneous tissue-like disorders (n=2561, ROR=0.68, PRR=0.69, χ2=369.34, IC=-0.53, EBGM=0.69) and neoplasms benign, malignant and unspecified (n=1047, ROR=0.56, PRR=0.56, χ2=364.75, IC=-0.83, EBGM=0.56). However, these signals did not meet positive thresholds across all four signal intensities, suggesting the need for further investigation.

## Signals detection based on preferred term levels

In our pharmacovigilance analysis, we identified 73 AEs categorised at the PT level in Table 2. We focused on PTs with more than 100 cases, as this is often a strong indication of a drug-related AE [29]. Key findings include reproductive system and breast disorders such as erectile dysfunction (n=3377), sexual dysfunction (n=2160) and ejaculatory dysfunction (n=652), and psychiatric disorders such as decreased sexual desire (n=1476). Notably, endocrine disorders such as hypogonadism (n=302) and post-5α-reductase inhibitor syndrome (n=166) were also identified, although these are not listed in the drug specifications and warrant further attention.

**Table 2. Signal strength of reports of finasteride administration at the preferred term level in the FAERS database**

| **PTs and categories** | **Case Reports** | **ROR(95% CI)** | **PRR(95% CI)** | **chisq** | **IC(IC025)** | **EBGM(EBGM05)** |
| --- | --- | --- | --- | --- | --- | --- |
| **reproductive system and breast disorders** |  |  |  |  |  |  |
| erectile dysfunction | 3377 | 144.15(138.85, 149.66) | 136.94(131.68, 142.41) | 385588.12 | 6.86(6.8) | 115.97(112.39) |
| sexual dysfunction | 2160 | 220.84(210.4, 231.79) | 213.75(205.53, 222.3) | 356099.85 | 7.38(7.31) | 166.6(159.99) |
| ejaculation disorder | 652 | 243.38(222.76, 265.92) | 241.02(222.85, 260.68) | 117980.64 | 7.51(7.39) | 182.7(169.65) |
| testicular pain | 618 | 223(203.8, 244) | 220.95(200.32, 243.7) | 104552.68 | 7.42(7.29) | 170.94(158.54) |
| ejaculation failure | 487 | 279.44(251.79, 310.14) | 277.42(251.52, 305.98) | 97945.39 | 7.66(7.52) | 202.84(185.9) |
| penile size reduced | 398 | 662.12(578.62, 757.67) | 658.19(573.81, 754.98) | 139167.42 | 8.46(8.28) | 351.19(313.73) |
| genital hypoaesthesia | 235 | 337.89(289.65, 394.18) | 336.71(287.84, 393.87) | 54305.2 | 7.86(7.65) | 232.77(204.62) |
| organic erectile dysfunction | 218 | 1094.72(889.08, 1347.92) | 1091.16(879.54, 1353.7) | 96782.38 | 8.8(8.56) | 445.36(374.2) |
| peyronie's disease | 215 | 286.11(244.49, 334.82) | 285.2(243.81, 333.62) | 44127.17 | 7.69(7.48) | 206.96(181.45) |
| testicular atrophy | 213 | 301.56(257.2, 353.56) | 300.6(256.98, 351.63) | 45419.19 | 7.75(7.53) | 214.94(188.15) |
| testicular disorder | 158 | 157.28(132.48, 186.73) | 156.91(131.53, 187.18) | 20246.36 | 7.02(6.78) | 129.96(112.58) |
| painful ejaculation | 96 | 707.65(535.46, 935.2) | 706.64(537.07, 929.76) | 34847.63 | 8.51(8.16) | 364.51(288.66) |
| varicocele | 89 | 461.45(354.37, 600.89) | 460.84(357.18, 594.58) | 25304.84 | 8.16(7.81) | 285.94(229.26) |
| male sexual dysfunction | 74 | 136.32(106.41, 174.65) | 136.17(105.54, 175.69) | 8405.1 | 6.85(6.5) | 115.42(93.81) |
| prostatic pain | 68 | 173.83(133.52, 226.32) | 173.65(134.59, 224.04) | 9480.26 | 7.14(6.77) | 141.22(113.25) |
| infertility male | 64 | 136.25(104.39, 177.84) | 136.12(103.46, 179.1) | 7266.87 | 6.85(6.47) | 115.38(92.33) |
| penile curvature | 63 | 328.78(244.5, 442.11) | 328.48(244.81, 440.75) | 14308.32 | 7.84(7.43) | 228.81(178.59) |
| male genital atrophy | 52 | 1116.34(727.21, 1713.69) | 1115.47(724.75, 1716.82) | 23293.43 | 8.81(8.32) | 449.35(313.93) |
| genital atrophy | 49 | 944(619.84, 1437.69) | 943.31(625.03, 1423.67) | 20441.53 | 8.71(8.21) | 418.62(294.41) |
| epididymal cyst | 49 | 800.35(535.17, 1196.92) | 799.77(529.92, 1207.04) | 18928.1 | 8.6(8.11) | 387.77(276.9) |
| genital paraesthesia | 49 | 230.1(167.07, 316.91) | 229.93(168.04, 314.62) | 8550.4 | 7.46(7.02) | 176.26(134.84) |
| scrotal disorder | 38 | 115.57(82.13, 162.64) | 115.51(82.78, 161.19) | 3738.49 | 6.65(6.16) | 100.24(75.32) |
| male reproductive tract disorder | 26 | 330.99(208.65, 525.06) | 330.86(206.71, 529.59) | 5935.04 | 7.85(7.22) | 229.96(156.31) |
| oligospermia | 23 | 239.92(150.02, 383.68) | 239.84(149.84, 383.9) | 4145.99 | 7.51(6.86) | 182.01(122.88) |
| spermatocele | 22 | 660.92(372.64, 1172.21) | 660.7(374.24, 1166.43) | 7708.26 | 8.46(7.74) | 351.91(217.87) |
| testicular cyst | 21 | 133.66(84.02, 212.63) | 133.62(83.48, 213.88) | 2346.52 | 6.83(6.18) | 113.58(77.02) |
| penile vascular disorder | 17 | 170.22(100.54, 288.2) | 170.18(100.25, 288.89) | 2330.86 | 7.12(6.39) | 138.92(89.42) |
| breast disorder male | 17 | 138.77(82.71, 232.83) | 138.73(83.34, 230.93) | 1962.06 | 6.87(6.15) | 117.25(76.05) |
| hypospermia | 16 | 750.98(375.54, 1501.75) | 750.8(378.09, 1490.91) | 5990.42 | 8.55(7.71) | 375.9(210.49) |
| feminisation acquired | 16 | 387.6(212, 708.66) | 387.51(211.06, 711.48) | 4068.39 | 8(7.2) | 255.93(154.48) |
| prostatic calcification | 16 | 130.6(76.8, 222.1) | 130.57(76.92, 221.65) | 1752.52 | 6.8(6.06) | 111.38(71.43) |
| genital disorder | 13 | 157.46(86.59, 286.31) | 157.43(85.75, 289.04) | 1670.38 | 7.03(6.2) | 130.31(79.01) |
| testis discomfort | 13 | 131.92(73.17, 237.86) | 131.9(73.26, 237.47) | 1436.42 | 6.81(6) | 112.34(68.6) |
| penile discomfort | 12 | 121.77(66.17, 224.12) | 121.75(66.31, 223.54) | 1236.59 | 6.71(5.87) | 104.9(62.97) |
| teratospermia | 10 | 208.59(103.51, 420.32) | 208.56(102.99, 422.35) | 1616.56 | 7.35(6.41) | 163.43(90.94) |
| prostate tenderness | 9 | 168.95(81.98, 348.2) | 168.93(81.8, 348.86) | 1226.47 | 7.11(6.13) | 138.09(75.4) |
| testicular microlithiasis | 6 | 250.29(99.35, 630.55) | 250.27(99.62, 628.75) | 1117.22 | 7.55(6.34) | 187.95(86.75) |
| prostatic atrophy | 5 | 250.29(90.96, 688.68) | 250.27(90.32, 693.49) | 931.02 | 7.55(6.25) | 187.95(80.58) |
| epididymal disorder | 4 | 176.67(59.44, 525.06) | 176.66(58.95, 529.44) | 565.58 | 7.16(5.76) | 143.2(57.56) |
| epididymal tenderness | 3 | 450.5(107.66, 1885.15) | 450.48(107.72, 1883.93) | 840.9 | 8.14(6.44) | 281.93(85.11) |
| oligoasthenozoospermia | 3 | 160.89(46.24, 559.89) | 160.89(45.89, 564.04) | 392.56 | 7.05(5.49) | 132.67(46.73) |
| **renal and urinary disorders** |  |  |  |  |  |  |
| terminal dribbling | 13 | 278.92(147.57, 527.2) | 278.87(148.94, 522.15) | 2624.52 | 7.67(6.81) | 203.61(119.52) |
| post micturition dribble | 6 | 136.52(57.2, 325.83) | 136.51(57.63, 323.37) | 682.93 | 6.85(5.7) | 115.66(55.86) |
| **endocrine disorders** |  |  |  |  |  |  |
| hypogonadism | 302 | 365.59(318.58, 419.54) | 363.95(317.29, 417.47) | 73623.19 | 7.94(7.75) | 245.45(218.75) |
| post 5-alpha-reductase inhibitor syndrome | 166 | 10411.85(5795.02, 18706.87) | 10386.07(5768.81, 18698.91) | 116208.64 | 9.45(9.15) | 701.12(429.4) |
| hypogonadism male | 79 | 418.19(317.58, 550.67) | 417.7(317.46, 549.59) | 21101.15 | 8.07(7.7) | 268.74(213.47) |
| androgen deficiency | 77 | 238.18(184.31, 307.8) | 237.91(184.4, 306.95) | 13794.31 | 7.5(7.14) | 180.9(145.97) |
| testicular failure | 72 | 1061.09(741.19, 1519.07) | 1059.95(744.85, 1508.36) | 31583.88 | 8.78(8.36) | 440.08(325.95) |
| secondary hypogonadism | 42 | 137.19(98.73, 190.63) | 137.1(98.25, 191.31) | 4798.42 | 6.86(6.39) | 116.09(88.15) |
| oestrogenic effect | 4 | 333.71(102.76, 1083.67) | 333.69(102.95, 1081.62) | 918.53 | 7.85(6.38) | 231.32(86.34) |
| **psychiatric disorders** |  |  |  |  |  |  |
| libido decreased | 1476 | 119.46(113.02, 126.26) | 116.85(110.18, 123.93) | 146728.83 | 6.66(6.58) | 101.25(96.66) |
| loss of libido | 1301 | 160.75(151.35, 170.73) | 157.65(148.65, 167.2) | 167389.74 | 7.03(6.94) | 130.46(124.05) |
| orgasm abnormal | 109 | 135.26(110.3, 165.88) | 135.04(111, 164.28) | 12291.97 | 6.84(6.55) | 114.61(96.62) |
| male orgasmic disorder | 108 | 240.29(193.46, 298.45) | 239.9(193.37, 297.62) | 19472.09 | 7.51(7.21) | 182.05(151.85) |
| premature ejaculation | 83 | 124.29(98.52, 156.8) | 124.14(98.12, 157.06) | 8699.7 | 6.74(6.41) | 106.67(87.82) |
| orgasmic sensation decreased | 74 | 228.89(176.43, 296.96) | 228.64(177.21, 294.99) | 12856.54 | 7.46(7.09) | 175.5(141.15) |
| psychogenic erectile dysfunction | 8 | 600.71(237.07, 1522.11) | 600.64(239.08, 1508.99) | 2660.63 | 8.38(7.24) | 334.13(153.48) |
| catastrophic reaction | 8 | 150.18(70.29, 320.85) | 150.16(69.92, 322.5) | 987.78 | 6.97(5.94) | 125.3(66.39) |
| psychophysiologic insomnia | 5 | 288.79(102.95, 810.09) | 288.77(102.19, 816.01) | 1035.57 | 7.71(6.38) | 208.83(88.1) |
| thermophobia | 3 | 150.17(43.47, 518.73) | 150.16(43.68, 516.2) | 370.42 | 6.97(5.41) | 125.3(44.41) |
| **congenital, familial and genetic disorders** |  |  |  |  |  |  |
| reproductive tract hypoplasia, male | 5 | 234.64(85.96, 640.53) | 234.63(86.35, 637.54) | 886.21 | 7.48(6.18) | 179(77.26) |
| androgen insensitivity syndrome | 4 | 375.42(113.04, 1246.79) | 375.4(113.57, 1240.9) | 995.74 | 7.97(6.48) | 250.6(91.79) |
| pseudohermaphroditism male | 3 | 2252.5(234.29, 21655.66) | 2252.4(236.45, 21455.83) | 1687.8 | 9.14(7.24) | 563.85(84.87) |
| **surgical and medical procedures** |  |  |  |  |  |  |
| hair transplant | 49 | 2832.01(1536.43, 5220.08) | 2829.94(1541.35, 5195.83) | 29054.83 | 9.21(8.68) | 594.16(356.19) |
| vasectomy | 31 | 173.77(117.57, 256.84) | 173.69(117.36, 257.05) | 4322.65 | 7.14(6.6) | 141.25(101.86) |
| radical prostatectomy | 15 | 154.31(88.52, 268.98) | 154.27(89.11, 267.07) | 1894.86 | 7(6.23) | 128.15(80.5) |
| varicocele repair | 8 | 2002.37(531.2, 7547.99) | 2002.13(528.04, 7591.37) | 4363.93 | 9.09(7.85) | 546.76(180.14) |
| radiotherapy to prostate | 8 | 333.73(145.1, 767.55) | 333.69(146.5, 760.07) | 1837.06 | 7.85(6.77) | 231.32(115.23) |
| scrotal operation | 6 | 409.56(151.46, 1107.5) | 409.53(150.72, 1112.77) | 1582.17 | 8.05(6.79) | 265.34(115.43) |
| penile prosthesis insertion | 6 | 128.72(54.14, 306.04) | 128.71(54.33, 304.89) | 649.04 | 6.78(5.63) | 110.02(53.3) |
| rectal fistula repair | 3 | 173.27(49.37, 608.06) | 173.26(49.42, 607.4) | 417.46 | 7.14(5.57) | 140.96(49.31) |
| uvulopalatopharyngoplasty | 3 | 125.14(36.86, 424.84) | 125.13(37.12, 421.81) | 316.65 | 6.75(5.21) | 107.4(38.62) |
| **nervous system disorders** |  |  |  |  |  |  |
| pudendal canal syndrome | 12 | 214.55(112.95, 407.55) | 214.51(112.34, 409.59) | 1983.51 | 7.38(6.51) | 167.07(97.66) |

ROR, reporting odds ratio; CI, confidence interval; PRR, proportional reporting ratio; chisq, chi-squared; IC, information component; EBGM, empirical Bayesian geometric mean; IC025, the lower limit of 95% CI of the IC; EBGM05, the lower limit of 95% CI of EBGM; PTs: preferred terms

In order to improve the stability of the calculations with fewer cases[30], we also a1nalyzed the IC values and found that male pseudohermaphroditism male (n= 3, IC= 9.14), varicocele repair (n= 8, IC= 9.09), psychogenic erectile dysfunction (n= 8, IC= 8.38), epididymal tenderness (n= 3, IC= 8.14), scrotal operation (n= 6, IC= 8.05) , and androgen insensitivity syndrome (n=4, IC=7.97) were among the unexpected signals with higher IC values. This implies a robust correlation with finasteride dosage.

To classify the early warning signals more carefully[31], we performed a descending order ranking of the BCPNN algorithm, and after excluding drug-unrelated signals, the top 10 finasteride PTs were post-5α reductase inhibitor syndrome (EBGM=701.12), hair transplant (EBGM=594.16), pseudohermaphroditism male (EBGM=563.85), varicocele repair (EBGM=546.76), male genital atrophy (EBGM=449.35), organic erectile dysfunction (EBGM=445.36), testicular failure (EBGM=440.08), genital atrophy (EBGM=418.62), epididymal cysts (EBGM=387.77), and hypospermia (EBGM= 375.9).

In summary, we found that ejaculation disorder, sexual dysfunction, ejaculation disorder, and epididymal tenderness were consistent with the drug insert and dosing warnings. Unexpectedly post-5α reductase inhibitor syndrome, hair transplant, male pseudohermaphroditism male, Peyronie's disease, male genital atrophy, epididymal cysts, testicular failure, varicocele repair, and androgen insensitivity syndrome were absent from the drug insert and require additional investigation.

## Time to Onset Analysis

Out of all AEs reported, after excluding inaccurate, missing, or unknown sex time of onset reports, a total of 7019 included the onset time, with a median onset time of 61 days (interquartile range 6.00-463.75). In Fig 4, it was shown that the time of onset in males (n=1208) versus females (n=47) was predominantly within one month after initiation of finasteride medication. Interestingly, after one year of finasteride treatment, AEs could still occur in males (n=879) and females (n=11). This indicates the importance of ongoing patient monitoring for potential AEs even beyond one year of finasteride treatment.

**Fig 4. Time to onset of AEs in male and female patients receiving finasteride therapy. AEs: adverse events.**

## Subgroup analysis

### Sex in different PT groups

The study investigated the effect of sex on adverse effects of finasteride treatment by analysing 102 male and 7 female AEs using four statistical methods, with the results detailed in S4-S5 Tables. Subgroup analyses showed that depression and anxiety were common AEs in both sexes. The 'volcano plot' in Fig 5 visualised the sex-specific signals, with blue dots indicating female-associated AEs and red dots indicating male-associated AEs. Cervical stenosis was more common in women, while post-micturition dribbling was more common in men. These findings highlight sex differences in AEs associated with finasteride treatment and underline the need for sex-specific clinical management.

**Fig 5. Sex-differentiated risk signal volcano plot for finasteride.**

The horizontal coordinate shows the log2 PRR value and the vertical coordinate indicates the adjusted p-value after -log10 conversion PRR, proportional reporting ratio.

### Age in different PT groups

Age is a known risk factor for AGA [32], with the incidence of AGA increasing with age [33]. To minimise the confounding effects of age on the study of adverse reactions to finasteride, we performed an age-stratified analysis.

We evaluated 17 adverse reactions in three age groups: patients aged <18 years, patients aged 18-65 years, and patients aged ≥65 years. The results, detailed in S6-S8 Tables, were arranged in descending order of the frequency of adverse reactions, and we excluded specific indications mentioned in the adverse reaction reports.In patients aged <18 years, the three most common adverse reactions were depression (n=11), erectile dysfunction (n=10) and cryptorchidism (n=8). In the 18-65 age group, the most common adverse reactions were erectile dysfunction (n=1435), depression (n=979) and anxiety (n=704). In patients aged ≥65 years, the top three AEs were depression (n=102), gynaecomastia (n=73) and erectile dysfunction (n=70). Common AEs across all age groups included erectile dysfunction, decreased libido, depression and suicidal ideation (S9 Table).

As most of the cases were less than 50 after subgroup analysis, we used IC values for descending order. The three most common AEs in patients aged <18 years based on IC values were scrotal disorders (IC=10.92), libido decreased (IC=9.38) and loss of libido (IC=9.01). In patients aged 18-65 years, the most common PTs were post-5-alpha reductase inhibitor syndrome (IC=9.55), male genital atrophy (IC=9.32), and genital atrophy (IC=8.89). In patients aged ≥65 years, the most common PTs were prostate tenderness (IC=9.98), penile shrinkage (IC=9.57), and penile exfoliation (IC=9.33). Finally, of all the PTs, the highest IC values in patients aged <18 years were observed for reproductive system and breast disorders. For patients aged 18-65 years, the highest IC values were found for endocrine disorders. In patients aged ≥65 years, reproductive system and breast disorders again had the highest IC values. Common PTs in all age groups included erectile dysfunction, libido decreased, depression, suicidal ideation, psychotic disorders and disturbance in attention. These findings suggest that different age groups experience different AEs, with a different focus on SOCs.

# Discussion

To our knowledge, earlier studies on finasteride safety have primarily been confined to clinical trials or concentrated on particular AEs, such as sexual dysfunction, and have mainly investigated potential safety concerns[34–36], whereas our study discusses this more fully and extensively. Although in a large real-world drug study in 2017 it comprehensively and systematically summarized global reports of finasteride-associated sexual dysfunction in FAERS, we offered a more precise and comprehensive description and identification of AEs associated with finasteride. Although the incidence of AEs reported in clinical trials was low, the risk of occurrence of finasteride-associated AEs still exists, necessitating further studies and additions to the literature.

With regard to ‘reproductive system and breast disorders’, sexual dysfunction is the most significant adverse effect of finasteride treatment in young men with AGA, manifested by loss of libido and erectile dysfunction[37]. Moreover, the distribution of medication-related AEs indicated that the highest number of cases (3146 27.22%) were in the age group of 18-45 years, and in the younger age group even a low dose of finasteride (1mg) may lead to persistent sexual dysfunction in young males, which may increase the risk of suicide [38]and persist after discontinuation of the medication[8]. This may be due to the fact that younger patients have more stressful lives, are more sensitive to hormonal changes than older adults [39], are more prone to symptoms, and are more susceptible to the adverse effects of finasteride. These two adverse effects further confirm the existing knowledge about the potential adverse consequences of finasteride on sexual functioning and the increased risk of suicide[40]. It was found that an increased risk of suicide may exist with finasteride in the treatment of men with a history of psychological disorders[41]. Surprisingly, the 2023 Treatment Update for AGA shows that suicide and depression in patients with AGA may be caused by sexual dysfunction (erectile dysfunction, hypospermia, neurological dysfunction), a side effect of finasteride (1mg once daily)[42]. Therefore, comprehensive and precise clinical screening for the drug and close monitoring of patients for post-drug adverse effects are warranted.

With regard to the ‘psychiatric disorders’, psychological distress in patients with AGA has become a significant motivator in the development of effective treatments and the reduction of adverse effects of medication[43]. There is a risk of suicide, depression, and anxiety in patients younger than 40 years of age treated with finasteride for alopecia areata[44], which may be related to the involvement of 5-ARIs in the central nervous system's synthesis of neuroactive steroidal organisms[45]. Neurosteroids are important physiological regulators of neural function in the adult brain and are involved in the control of the neuroendocrine system of reproduction[46]. A small human study observed depressive symptoms associated with reduced levels of neurosteroids in the cerebrospinal fluid of men who were taking finasteride[47]. This observation was supported by animal studies[48,49]. A study has been conducted by evaluating FMRI and HPLC-mass spectrometry analyses of neurosteroid levels in cerebrospinal fluid, pointing to the fact that finasteride disrupts neurotransmitters and chemical messengers[50]. These findings reinforce that anxiety and depression induced by finasteride use cannot be ignored. However, it has been suggested that the results of disproportionate analyses of AE signals may be influenced by the susceptibility of young patients to adverse factors, and further sensitivity analyses have shown an association between AGA and psychological AEs in young people that is not due to drug use[51]. This is consistent with the fact that pharmacovigilance does not directly prove biological causation. Emerging evidence also suggests that finasteride inhibits glioblastoma proliferation[52]. These findings highlight the potential relevance of finasteride to neuroscience and the need to closely monitor neurological psychiatric problems in patients receiving finasteride.

With regard to ‘renal and urinary disorders’, the only PT's with positive signals were post micturition dribble and terminal dribbling, the former of which is the most frequent adverse reaction in men. Traish suggests that the symptoms are due to a novel tissue-specific androgen deficiency that develops when finasteride inhibits the enzyme 5-alpha reductase, which reduces the biosynthesis of 5alpha-dihydrotestosterone[53]. The symptoms of post micturition dribble have been shown to be associated with the development of a new form of androgen deficiency in men. In addition, finasteride has been widely used in the treatment of a variety of testosterone-related disorders, and new evidence suggests that finasteride has a protective effect on testosterone-induced calcium oxalate crystals, which is effective in preventing the formation of calcium oxalate-type renal stones and neutralising the calcium oxalate-promoting effects of testosterone[54]. Future studies could explore the relationship between finasteride and testosterone levels, and reduce the prevalence of renal stones in patients with high levels of the testosterone.

At the PT level, we discovered that while prostate pain, prostate calcification, and prostate atrophy were notable in disproportionate assessments and identified as adverse reactions by certain studies, they were regarded as treatment indications in Table 1, consistent with findings from another research. Although finasteride reduces the risk of prostate cancer, it increases the malignancy of high-grade prostate cancer[55]. To ensure the reliability of our results, we omitted adverse reactions listed as indications in Table 2.

In addition to the expected AEs associated with finasteride use, our study identified a number of unintended AEs not mentioned in the specification that need to be further analyzed and evaluated, including ‘Peyronie's disease’, ‘post-5α reductase inhibitor syndrome’, ‘catastrophic reaction’ and “thermophobia”. Peyronie's disease is a male condition characterized by penile curvature, pain, penile shortening and erectile dysfunction[56]. This is an unintended AE associated with AGA treated with finasteride (176/210 83.8%) [57]and the results of our study are consistent with the above studies. In addition, a case report found that the pathogenesis of finasteride-treated AGA is the same as that of Peyronie's disease, in that inflammation affects the penile leukomalacia, leading to the formation of inelastic microtissue that deforms the penis[58,59]. However, the relationship needs to be further determined[57]. Another specification unmentionable AE that appeared in our analysis was the post 5-alpha-reductase inhibitor syndrome, 5-ARIs are drugs used to treat androgen-dependent diseases. Long-term use of 5-ARIs in patients with AGA leads to persistent side effects known as post-finasteride syndrome (PFS) [60,61]. PFS is also characterized by symptoms of penile shortening and penile curvature[62]. Not only this, but also persistent or irreversible sexual, neurological, physical and psychiatric side effects[63]. Leliefeld et al. have demonstrated that finasteride, a 5α-reductase inhibitor, inhibits the conversion of testosterone to 5α dihydrotestosterone (DHT) in the prostate and scalp and impairs the levels of other neurosteroids in the brain, demonstrating that sustained changes in androgen receptor and neurosteroid levels are associated with PFS[64]. However, previous studies have been inconclusive as to whether or not PFS exists[65]. Finally, our study reinforces the need for additional clinical research to assess the balance between the health risks and benefits of the medication. Careful consideration is required when using finasteride and developing potential new therapeutic options for treating these two conditions.

There is no mention of the adverse reaction in the insert: male breast cancer in the FAERS database or in the previous literature. As for the rare adverse reaction in the specification: hypersensitivity reaction, there was one case report of a patient with prostatic hyperplasia who developed maculopapular rash and rash on the extremities after 2 months of drug administration[66]. According to the FAERS database, SOCs not mentioned in the specification included endocrine system diseases, renal and urinary system diseases, various surgical and medical operations, and various congenital familial hereditary diseases. It should be noted that we conducted a revalidation of the results of the specification from a pharmacovigilance perspective for reproductive and breast disorders, psychiatric categories, and various neurological disorders. There is emerging evidence that finasteride both treats suppurative sweats in women[67] and reduces the incidence of bladder cancer[68,69]and induces type 2 diabetes[70], down-regulates androgen receptor expression in the renal cortex[71], forms blood clots[72], and alters intestinal microbial abundance[73,73,74] among other things. With regard to ‘endocrine disorders’ and ‘renal and urinary disorders’ it is noteworthy that almost all clinical trials did not include primary and secondary endpoint sensitisers for type 2 diabetes mellitus, renal dysfunction indicators. These unintended AEs raise concerns about the effects of finasteride on endocrine and renal disorders and the potential risk of severe endocrine disorders[75]. Further studies are necessary to investigate the prevalence of hormonal and metabolic changes and to evaluate the level of risk for individuals taking finasteride.

With respect to the timing of AEs and final outcome, the probability of AEs was greatest on days 0-31 of finasteride use in our analysis. Therefore, it is important to closely monitor potential risks associated with medication use over a one-month period. However, it is also possible that patients linked and reported their medication side effects within a short period of time after using the drug, which could also lead to bias in the data set. In addition, disability and life-threatening events such as male reproductive tract hypoplasia and catastrophic reactions were also reported in our study, suggesting that comprehensive monitoring and management of AEs receiving finasteride medication should not be underestimated, especially in the treatment of male patients with AGA and prostatic hyperplasia[76].

From the perspective of sex subgroups, male patients reported a higher incidence of AEs with finasteride compared to female patients. This sex difference can be understood from both sociological and biological perspectives. From the sociological point of view, firstly, the prevalence of AGA is on the rise globally, with 45.72% of men and 5.05% of women[77]. Furthermore, premenopausal women suffering from hair loss are mainly treated with minoxidil, oral contraceptives, and cosmetic products[78]. Finasteride, on the other hand, has been used to promote hair growth mainly after menopause[79,80]. As a result, the population of women who use finasteride is relatively small. Secondly, women prefer hair transplants and cosmetics to increase scalp hair fullness[81]. Finally, the safety of oral finasteride in patients with a history of gynaecological malignancy has not yet been clarified, which makes the use of this drug in the treatment of AGA more limited, especially when compared to male patients[82]. Biologically, AGA, previously thought to have an autosomal dominant mode of inheritance, has a reduced epistasis in females[83],this makes males more susceptible to AGA than females[84]. Furthermore, female follicle levels of type I and type II 5α-reductase and androgen receptors are half those of males. The percentage of females with a clinical diagnosis of AGA is then lower than that of males. In summary, we hypothesize that men experience more AEs than women because of the aforementioned biological and sociological factors, and further investigation into the underlying mechanisms and reasons is warranted.

At the age subgroup level, our study found that patients aged 18-65 years were more likely to report AEs involving finasteride than patients aged <18 years and those patients aged ≥65 years. Because of the high correlation between AGA severity and age factors[85], AGA not only affects social functioning and emotional well-being in young men[86], but also affects sexual functioning and reduces quality of life in women aged 18-65 years[87]. Therefore, clinicians can kindly remind patients aged 18-65 years of the medication precautions when using it in the clinical setting, so that finasteride AEs will not go unnoticed in the non-medical setting.

# Limitation

It is crucial to recognize some constraints of our research, primarily arising from the fundamental features of pharmacovigilance databases. Firstly, up to 53.67% of the reports in our study originated from consumers (n=6203), given the potential for inaccurate or misleading content in reports submitted by non-medical professionals due to limitations in their medical knowledge. Second, our data analyses failed to cover a wide range of unquantified confounding variables that may affect AEs, such as potential drug interactions, changes in treatment regimens, and results of laboratory tests and instrumental examinations. Additionally, the FAERS database is subject to incomplete or inaccurate data, particularly regarding drug therapy duration. Missing information on treatment cessation and adherence may impact the timing of AEs. Finally, further prospective studies are needed to confirm and clarify the relationship between finasteride and these AEs, addressing issues like data inconsistency and confounding factors.

# Conclusions

We performed pharmacovigilance analyses using actual data from the FAERS database, and the adverse reactions identified in the survey were generally consistent with those in the package inserts. Additionally, we detected AEs not listed in the specifications, including post 5-alpha-reductase inhibitor syndrome, Peyronie's disease, testicular microlithiasis, and catastrophic reactions. The identification of these strongly indicating AEs supplements the inherent limitation of the relatively small sample size in clinical studies of this drug. The results of this study help to inform the safe and rational use of the drug in the clinic.

# Acknowledgments

We would like to thank the participants and researchers of the FAERS database. We also acknowledge Medex UIMA 1.8.3 system for providing their platforms and contributors for uploading meaningful data.

# References

1.	Adil A, Godwin M. The effectiveness of treatments for androgenetic alopecia: A systematic review and meta-analysis. J Am Acad Dermatol. 2017;77: 136-141.e5. doi:10.1016/j.jaad.2017.02.054

2.	Lauck KC, Limmer A, Harris P, Kivelevitch D. Sexual dysfunction with 5-alpha-reductase inhibitor therapy for androgenetic alopecia: A global propensity score matched retrospective cohort study. J Am Acad Dermatol. 2024;91: 163–166. doi:10.1016/j.jaad.2024.03.019

3.	Enitan A, Olasode O, Onayemi O, Ajani A, Oninla O, Olanrewaju F, et al. Androgenetic alopecia: What impact does it have on the quality of Life? Niger J Med. 2022;31: 455. doi:10.4103/NJM.NJM_61_22

4.	Ntshingila S, Oputu O, Arowolo AT, Khumalo NP. Androgenetic alopecia: An update. JAAD Int. 2023;13: 150–158. doi:10.1016/j.jdin.2023.07.005

5.	Asfour L, Cranwell W, Sinclair R. Male Androgenetic Alopecia. In: Feingold KR, Anawalt B, Blackman MR, Boyce A, Chrousos G, Corpas E, et al., editors. Endotext. South Dartmouth (MA): MDText.com, Inc.; 2000. Available: http://www.ncbi.nlm.nih.gov/books/NBK278957/

6.	Piraccini BM, Blume‐Peytavi U, Scarci F, Jansat JM, Falqués M, Otero R, et al. Efficacy and safety of topical finasteride spray solution for male androgenetic alopecia: a phase III, randomized, controlled clinical trial. J Eur Acad Dermatol Venereol. 2022;36: 286–294. doi:10.1111/jdv.17738

7.	Xiao Q, Wang L, Supekar S, Shen T, Liu H, Ye F, et al. Structure of human steroid 5α-reductase 2 with the anti-androgen drug finasteride. Nat Commun. 2020;11: 5430. doi:10.1038/s41467-020-19249-z

8.	Traish AM. Post-finasteride syndrome: a surmountable challenge for clinicians. Fertil Steril. 2020;113: 21–50. doi:10.1016/j.fertnstert.2019.11.030

9.	Garcia-Argibay M, Hiyoshi A, Fall K, Montgomery S. Association of 5α-Reductase Inhibitors With Dementia, Depression, and Suicide. JAMA Netw Open. 2022;5: e2248135. doi:10.1001/jamanetworkopen.2022.48135

10.	Traish AM. The Post-finasteride Syndrome: Clinical Manifestation of Drug-Induced Epigenetics Due to Endocrine Disruption. Curr Sex Health Rep. 2018;10: 88–103. doi:10.1007/s11930-018-0161-6

11.	Traish AM, Melcangi RC, Bortolato M, Garcia-Segura LM, Zitzmann M. Adverse effects of 5α-reductase inhibitors: What do we know, don’t know, and need to know? Rev Endocr Metab Disord. 2015;16: 177–198. doi:10.1007/s11154-015-9319-y

12.	Merck Supplementary file.

13.	Irwig MS. How routine pharmacovigilance failed to identify finasteride’s persistent sexual side effects. Andrology. 2022;10: 207–208. doi:10.1111/andr.13122

14.	Li D, Chai S, Wang H, Dong J, Qin C, Du D, et al. Drug-induced QT prolongation and torsade de pointes: a real-world pharmacovigilance study using the FDA Adverse Event Reporting System database. Front Pharmacol. 2023;14: 1259611. doi:10.3389/fphar.2023.1259611

15.	Nagai J, Ishikawa Y. Analysis of anticholinergic adverse effects using two large databases: The US Food and Drug Administration Adverse Event Reporting System database and the Japanese Adverse Drug Event Report database. Yang J-M, editor. PLOS ONE. 2021;16: e0260980. doi:10.1371/journal.pone.0260980

16.	Yu RJ, Krantz MS, Phillips EJ, Stone CA. Emerging Causes of Drug-Induced Anaphylaxis: A Review of Anaphylaxis-Associated Reports in the FDA Adverse Event Reporting System (FAERS). J Allergy Clin Immunol Pract. 2021;9: 819-829.e2. doi:10.1016/j.jaip.2020.09.021

17.	Shu Y, Ding Y, Liu Y, Wu P, He X, Zhang Q. Post-Marketing Safety Concerns With Secukinumab: A Disproportionality Analysis of the FDA Adverse Event Reporting System. Front Pharmacol. 2022;13: 862508. doi:10.3389/fphar.2022.862508

18.	Yang X, Yang B, Li D, Pan W, Tong Q, Wang L, et al. Thromboembolic Events Associated with Epidermal Growth Factor Receptor Tyrosine Kinase Inhibitors: A Pharmacovigilance Analysis of the US FDA Adverse Event Reporting System (FAERS) Database. Clin Drug Investig. 2024;44: 199–207. doi:10.1007/s40261-024-01346-2

19.	Liu N, Liang X, Chen Y, Xie L. Recent trends in treatment strategies for Alzheimer’s disease and the challenges: A topical advancement. Ageing Res Rev. 2024;94: 102199. doi:10.1016/j.arr.2024.102199

20.	Liu M, Gu L, Zhang Y, Zhou H, Wang Y, Xu Z-X. A real-world disproportionality analysis of mesalazine data mining of the public version of FDA adverse event reporting system. Front Pharmacol. 2024;15: 1290975. doi:10.3389/fphar.2024.1290975

21.	Liu M, Gu L, Zhang Y, Zhou H, Wang Y, Xu Z-X. A real-world disproportionality analysis of mesalazine data mining of the public version of FDA adverse event reporting system. Frontiers In Pharmacology. Key Laboratory of Pathobiology, Ministry of Education, Jilin University, Changchun, Jilin, China.; 2024. p. 1290975. doi:10.3389/fphar.2024.1290975

22.	Zou F, Cui Z, Lou S, Ou Y, Zhu C, Shu C, et al. Adverse drug events associated with linezolid administration: a real-world pharmacovigilance study from 2004 to 2023 using the FAERS database. Frontiers In Pharmacology. Department of Tuberculosis, Affiliated Hospital of Zunyi Medical University, Zunyi, Guizhou, China.; 2024. p. 1338902. doi:10.3389/fphar.2024.1338902

23.	Hung E, Hauben M, Essex H, Zou C, Bright S. More extreme duplication in FDA Adverse Event Reporting System detected by literature reference normalization and fuzzy string matching. Pharmacoepidemiol Drug Saf. 2023;32: 387–391. doi:10.1002/pds.5555

24.	Zou F, Cui Z, Lou S, Ou Y, Zhu C, Shu C, et al. Adverse drug events associated with linezolid administration: a real-world pharmacovigilance study from 2004 to 2023 using the FAERS database. Front Pharmacol. 2024;15: 1338902. doi:10.3389/fphar.2024.1338902

25.	Bao Y, Chen J, Duan L, Wang F, Lai H, Mo Z, et al. Comparing the difference of adverse events with HER2 inhibitors: a study of the FDA adverse event reporting system (FAERS). Front Pharmacol. 2024;15: 1288362. doi:10.3389/fphar.2024.1288362

26.	Wu T, Shi Y, Zhu B, Li D, Li Z, Zhao Z, et al. Pregnancy-related adverse events associated with statins: a real-world pharmacovigilance study of the FDA Adverse Event Reporting System (FAERS). Expert Opin Drug Saf. 2024;23: 313–321. doi:10.1080/14740338.2023.2251888

27.	Wu B, Shen P, Yin X, Yu L, Wu F, Chen C, et al. Analysis of adverse event of interstitial lung disease in men with prostate cancer receiving hormone therapy using the Food and Drug Administration Adverse Event Reporting System. Br J Clin Pharmacol. 2023;89: 440–448. doi:10.1111/bcp.15336

28.	Wang G, Wang J, Du R, Wang Y, Li Z. Toxicity Spectrum of Anti-GD2 Immunotherapy: A Real-World Study Leveraging the US Food and Drug Administration Adverse Event Reporting System. Pediatr Drugs. 2023 [cited 10 Jul 2024]. doi:10.1007/s40272-023-00613-7

29.	Yu X, Zhou X, Li M, Zhao Y. Hematological adverse events associated with anti-MRSA agents: a real-world analysis based on FAERS. Expert Opin Drug Saf. 2024; 1–11. doi:10.1080/14740338.2024.2309225

30.	Zou F, Cui Z, Lou S, Ou Y, Zhu C, Shu C, et al. Adverse drug events associated with linezolid administration: a real-world pharmacovigilance study from 2004 to 2023 using the FAERS database. Front Pharmacol. 2024;15: 1338902. doi:10.3389/fphar.2024.1338902

31.	Du Y, Zhu J, Guo Z, Wang Z, Wang Y, Hu M, et al. Metformin adverse event profile: a pharmacovigilance study based on the FDA Adverse Event Reporting System (FAERS) from 2004 to 2022. Expert Rev Clin Pharmacol. 2024;17: 189–201. doi:10.1080/17512433.2024.2306223

32.	Vinay K, Bhattachajee R, Bishnoi A, Kaushik A, Sachdeva N, Pal A, et al. Clinical and metabolic characteristics of males with early-onset androgenetic alopecia. Indian J Dermatol Venereol Leprol. 2022;89: 530–535. doi:10.25259/IJDVL_949_2021

33.	Liu L-P, Wariboko MA, Hu X, Wang Z-H, Wu Q, Li Y-M. Factors associated with early-onset androgenetic alopecia: A scoping review. Jayakody S, editor. PLOS ONE. 2024;19: e0299212. doi:10.1371/journal.pone.0299212

34.	Kiguradze T, Temps WH, Yarnold PR, Cashy J, Brannigan RE, Nardone B, et al. Persistent erectile dysfunction in men exposed to the 5α-reductase inhibitors, finasteride, or dutasteride. PeerJ. 2017;5: e3020. doi:10.7717/peerj.3020

35.	Haber RS, Gupta AK, Epstein E, Carviel JL, Foley KA. Finasteride for androgenetic alopecia is not associated with sexual dysfunction: a survey-based, single-centre, controlled study. J Eur Acad Dermatol Venereol JEADV. 2019;33: 1393–1397. doi:10.1111/jdv.15548

36.	Giatti S, Diviccaro S, Cioffi L, Cosimo Melcangi R. Post-Finasteride Syndrome And Post-Ssri Sexual Dysfunction: Two Clinical Conditions Apparently Distant, But Very Close. Front Neuroendocrinol. 2024;72: 101114. doi:10.1016/j.yfrne.2023.101114

37.	Lauck KC, Limmer A, Harris P, Kivelevitch D. Sexual dysfunction with 5-alpha-reductase inhibitor therapy for androgenetic alopecia: A global propensity score matched retrospective cohort study. J Am Acad Dermatol. 2024;91: 163–166. doi:10.1016/j.jaad.2024.03.019

38.	Ali AK, Heran BS, Etminan M. Persistent Sexual Dysfunction and Suicidal Ideation in Young Men Treated with Low‐Dose Finasteride: A Pharmacovigilance Study. Pharmacother J Hum Pharmacol Drug Ther. 2015;35: 687–695. doi:10.1002/phar.1612

39.	Nguyen D-D, Marchese M, Cone EB, Paciotti M, Basaria S, Bhojani N, et al. Investigation of Suicidality and Psychological Adverse Events in Patients Treated With Finasteride. JAMA Dermatol. 2021;157: 35. doi:10.1001/jamadermatol.2020.3385

40.	Irwig MS, Sanz J, Lin D, Tan N, Dommasch E. Beliefs and counseling practices among dermatologists regarding sexual and other adverse effects of finasteride. Int J Impot Res. 2023. doi:10.1038/s41443-023-00750-0

41.	Laanani M, Weill A, Jollant F, Zureik M, Dray-Spira R. Suicidal risk associated with finasteride versus dutasteride among men treated for benign prostatic hyperplasia: nationwide cohort study. Sci Rep. 2023;13: 5308. doi:10.1038/s41598-023-32356-3

42.	Devjani S, Ezemma O, Kelley KJ, Stratton E, Senna M. Androgenetic Alopecia: Therapy Update. Drugs. 2023;83: 701–715. doi:10.1007/s40265-023-01880-x

43.	Frith H, Jankowski GS. Psychosocial impact of androgenetic alopecia on men: A systematic review and meta-analysis. Psychol Health Med. 2024;29: 822–842. doi:10.1080/13548506.2023.2242049

44.	Irwig MS. Finasteride and Suicide: A Postmarketing Case Series. Dermatology. 2020;236: 540–545. doi:10.1159/000505151

45.	Giatti S, Diviccaro S, Panzica G, Melcangi RC. Post-finasteride syndrome and post-SSRI sexual dysfunction: two sides of the same coin? Endocrine. 2018;61: 180–193. doi:10.1007/s12020-018-1593-5

46.	Micevych P, Sinchak K. The Neurosteroid Progesterone Underlies Estrogen Positive Feedback of the LH Surge. Front Endocrinol. 2011;2. doi:10.3389/fendo.2011.00090

47.	Melcangi RC, Caruso D, Abbiati F, Giatti S, Calabrese D, Piazza F, et al. Neuroactive steroid levels are modified in cerebrospinal fluid and plasma of post-finasteride patients showing persistent sexual side effects and anxious/depressive symptomatology. J Sex Med. 2013;10: 2598–2603. doi:10.1111/jsm.12269

48.	Howell S, Song W, Pastuszak A, Khera M. Differential Gene Expression in Post-Finasteride Syndrome Patients. J Sex Med. 2021;18: 1479–1490. doi:10.1016/j.jsxm.2021.05.009

49.	Giatti S, Cioffi L, Diviccaro S, Piazza R, Melcangi RC. Analysis of the finasteride treatment and its withdrawal in the rat hypothalamus and hippocampus at whole-transcriptome level. J Endocrinol Invest. 2024 [cited 4 Jul 2024]. doi:10.1007/s40618-024-02345-y

50.	Uleri A, Cornu JN, Gobbo A, Herrmann TRW, De Nunzio C, Hashim H, et al. Association of 5α-Reductase Inhibitors with Depression and Suicide: A Mini Systematic Review and Meta-analysis. Eur Urol Focus. 2024; S2405-4569(24)00062–2. doi:10.1016/j.euf.2024.04.009

51.	Choi JW, Huh CH, Choi GS. Association of Hair Loss With Suicidality and Psychological Adverse Events vs Finasteride Use. JAMA Dermatol. 2021;157: 737. doi:10.1001/jamadermatol.2021.1001

52.	Kim HJ, Kim T-J, Kim YG, Seong C, Cho J-H, Kim W, et al. Antioxidant and Antiproliferative Activity of Finasteride against Glioblastoma Cells. Pharmaceutics. 2021;13: 1410. doi:10.3390/pharmaceutics13091410

53.	Traish AM. Health Risks Associated with Long-Term Finasteride and Dutasteride Use: It’s Time to Sound the Alarm. World J Mens Health. 2020;38: 323. doi:10.5534/wjmh.200012

54.	Sueksakit K, Thongboonkerd V. Protective effects of finasteride against testosterone-induced calcium oxalate crystallization and crystal-cell adhesion. JBIC J Biol Inorg Chem. 2019;24: 973–983. doi:10.1007/s00775-019-01692-z

55.	Wang L, Lei Y, Gao Y, Cui D, Tang Q, Li R, et al. Association of finasteride with prostate cancer: A systematic review and meta-analysis. Medicine (Baltimore). 2020;99: e19486. doi:10.1097/MD.0000000000019486

56.	Paulis G, De Giorgio G, Paulis A. Clinical Presentation of Peyronie’s Disease: A Retrospective Study of 564 Cases. Diagn Basel Switz. 2024;14: 1125. doi:10.3390/diagnostics14111125

57.	Schifano N, Capogrosso P, Boeri L, Fallara G, Chiappini S, Rewhorn M, et al. Are finasteride-related penile curvature/Peyronie’s disease Adverse Event Reports worthy of further clinical investigation? Disproportionality analysis based on both the Food and Drug Administration (FDA) and the European Medicines Agency (EMA) pharmacovigilance databases. Int J Impot Res. 2023;35: 465–471. doi:10.1038/s41443-022-00568-2

58.	Paulis G, De Giorgio G, Paulis A. Clinical Presentation of Peyronie’s Disease: A Retrospective Study of 564 Cases. Diagnostics. 2024;14: 1125. doi:10.3390/diagnostics14111125

59.	Garreton AS, Valzacchi GR, Layus O. Post-Finasteride Syndrome: About 2 Cases and Review of the Literature. Androl-Open Access. 2016;05. doi:10.4172/2472-1212.1000170

60.	Traish AM. Post-finasteride syndrome: a surmountable challenge for clinicians. Fertil Steril. 2020;113: 21–50. doi:10.1016/j.fertnstert.2019.11.030

61.	Pereira AFJR, Coelho TO de A. Post-finasteride syndrome. An Bras Dermatol. 2020;95: 271–277. doi:10.1016/j.abd.2020.02.001

62.	Ganzer CA, Jacobs AR, Iqbal F. Persistent Sexual, Emotional, and Cognitive Impairment Post-Finasteride: A Survey of Men Reporting Symptoms. Am J Mens Health. 2015;9: 222–228. doi:10.1177/1557988314538445

63.	Traish AM. Post-finasteride syndrome: a surmountable challenge for clinicians. Fertil Steril. 2020;113: 21–50. doi:10.1016/j.fertnstert.2019.11.030

64.	Leliefeld HHJ, Debruyne FMJ, Reisman Y. The post-finasteride syndrome: possible etiological mechanisms and symptoms. Int J Impot Res. 2023 [cited 4 Jul 2024]. doi:10.1038/s41443-023-00759-5

65.	Li X, Guo Y, Lu Y, Li H, Yan S, Li H, et al. Case report: a study of the clinical characteristics and genetic variants of post-finasteride syndrome patients. Transl Androl Urol. 2022;11: 1452–1457. doi:10.21037/tau-22-92

66.	Jia H, Ran L. Maculopapular Drug Eruption Caused by Finasteride: A Case Report. Clin Cosmet Investig Dermatol. 2023;Volume 16: 3359–3361. doi:10.2147/CCID.S426747

67.	Babbush KM, Andriano TM, Cohen SR. Antiandrogen therapy in hidradenitis suppurativa: finasteride for females. Clin Exp Dermatol. 2022;47: 86–92. doi:10.1111/ced.14847

68.	Garg H, Wheeler KM, Dursun F, Cooper RE, Pruthi DK, Kaushik D, et al. Impact of Finasteride on Survival in Bladder Cancer: A Retrospective Multi-institutional Database Analysis. Clin Genitourin Cancer. 2023;21: 314.e1-314.e7. doi:10.1016/j.clgc.2022.10.014

69.	Zhu D, Srivastava A, Agalliu I, Fram E, Kovac EZ, Aboumohamed A, et al. Finasteride Use and Risk of Bladder Cancer in a Multiethnic Population. J Urol. 2021;206: 15–21. doi:10.1097/JU.0000000000001694

70.	Wei L, Lai EC-C, Kao-Yang Y-H, Walker BR, MacDonald TM, Andrew R. Incidence of type 2 diabetes mellitus in men receiving steroid 5α-reductase inhibitors: population based cohort study. BMJ. 2019;365: l1204. doi:10.1136/bmj.l1204

71.	Baig MS, Kolasa-Wołosiuk A, Pilutin A, Safranow K, Baranowska-Bosiacka I, Kabat-Koperska J, et al. Finasteride-Induced Inhibition of 5α-Reductase Type 2 Could Lead to Kidney Damage-Animal, Experimental Study. Int J Environ Res Public Health. 2019;16: 1726. doi:10.3390/ijerph16101726

72.	Gaddameedi SR, Khan MA, Arty F, Bandari V, Vangala A, Panchal P, et al. An Unusual Case of Thalamic Stroke in a Young Adult With Patent Foramen Ovale and Finasteride Use. Cureus. 2024 [cited 13 Jul 2024]. doi:10.7759/cureus.60300

73.	Borgo F, Macandog AD, Diviccaro S, Falvo E, Giatti S, Cavaletti G, et al. Alterations of gut microbiota composition in post-finasteride patients: a pilot study. J Endocrinol Invest. 2021;44: 1263–1273. doi:10.1007/s40618-020-01424-0

74.	An J, Song Y, Kim S, Kong H, Kim K. Alteration of Gut Microbes in Benign Prostatic Hyperplasia Model and Finasteride Treatment Model. Int J Mol Sci. 2023;24: 5904. doi:10.3390/ijms24065904

75.	Vinay K, Bhattachajee R, Bishnoi A, Kaushik A, Sachdeva N, Pal A, et al. Clinical and metabolic characteristics of males with early-onset androgenetic alopecia. Indian J Dermatol Venereol Leprol. 2022;89: 530–535. doi:10.25259/IJDVL_949_2021

76.	Xiao R, Lee LN. Updated Review of Treatment of Androgenetic Alopecia. Facial Plast Surg Clin N Am. 2024;32: 417–423. doi:10.1016/j.fsc.2024.02.006

77.	Zhu H, Guo H, Gao Y, Wei Y, Mao T, Yang J. A community-oriented survey on the association between androgenetic alopecia and metabolic syndrome in Chinese people. Front Med. 2022;9: 1009578. doi:10.3389/fmed.2022.1009578

78.	Pindado-Ortega C, Saceda-Corralo D, Fernández-Nieto D, Jiménez-Cauhé J, Ortega-Quijano D, Moreno-Arrones ÓM, et al. Prescribing Habits for Androgenic Alopecia among Dermatologists in Spain in 2019–2020: A Cross-Sectional Study. Skin Appendage Disord. 2020;6: 283–286. doi:10.1159/000509063

79.	Iamsumang W, Leerunyakul K, Suchonwanit P. Finasteride and Its Potential for the Treatment of Female Pattern Hair Loss: Evidence to Date. Drug Des Devel Ther. 2020;14: 951–959. doi:10.2147/DDDT.S240615

80.	Price VH, Roberts JL, Hordinsky M, Olsen EA, Savin R, Bergfeld W, et al. Lack of efficacy of finasteride in postmenopausal women with androgenetic alopecia. J Am Acad Dermatol. 2000;43: 768–776. doi:10.1067/mjd.2000.107953

81.	Sinclair R, Patel M, Dawson TL, Yazdabadi A, Yip L, Perez A, et al. Hair loss in women: medical and cosmetic approaches to increase scalp hair fullness: Approaches to increase scalp hair fullness. Br J Dermatol. 2011;165: 12–18. doi:10.1111/j.1365-2133.2011.10630.x

82.	Seyed Jafari SM, Heidemeyer K, Hunger RE, De Viragh PA. Safety of Antiandrogens for the Treatment of Female Androgenetic Alopecia with Respect to Gynecologic Malignancies. J Clin Med. 2024;13: 3052. doi:10.3390/jcm13113052

83.	Osborn D. INHERITANCE OF BALDNESS. J Hered. 1916;7: 347–355. doi:10.1093/oxfordjournals.jhered.a110746

84.	Sawaya ME, Price VH. Different Levels of 5α-Reductase Type I and II, Aromatase, and Androgen Receptor in Hair Follicles of Women and Men with Androgenetic Alopecia. J Invest Dermatol. 1997;109: 296–300. doi:10.1111/1523-1747.ep12335779

85.	Oiwoh S, Akinboro A, Olayemi O, Salawu A, Olasode O, Onayemi E. Androgenetic Alopecia: Traditional Cardiovascular Risk Factors, Metabolic Syndrome, and Component Traits among Nigerian Adults. Niger J Clin Pract. 2023;26: 463–469. doi:10.4103/njcp.njcp_530_22

86.	Adamowicz R, Załęcki P, Dukiel A, Nowicka D. Association between Androgenetic Alopecia and Psychosocial Disease Burden: A Cross-Sectional Survey among Polish Men. Burkhart CG, editor. Dermatol Res Pract. 2022;2022: 1–6. doi:10.1155/2022/1845044

87.	Krefft-Trzciniecka K, Cisoń H, Pakiet A, Nowicka D, Szepietowski JC. Enhancing Quality of Life and Sexual Functioning in Female Androgenetic Alopecia: Therapeutic Potential of Hair Follicle-Derived Stem Cells. Healthcare. 2024;12: 608. doi:10.3390/healthcare12060608

# Supporting information

**S1 Table. Four grid table.**

**S2 Table. Four major algorithms used for signal detection.**

**S3 Table. Signal strength of reports of finasteride administration at the SOC level**

**S4 Table. Signal strength of reports of finasteride administration in female at the PT level.**

**S5 Table. Signal strength of reports of finasteride administration in male at the PT level.**

**S6 Table. Signal strength of reports of finasteride administration at the PT level (age<18).**

**S7 Table. Signal strength of reports of finasteride administration at the PT level (age=18-65).**

**S8 Table. Signal strength of reports of finasteride administration at the PT level (age >=65).**

**S9 Table. Number of adverse reaction case reports of finasteride administration at the PT level.**
