# Supplementary material for: Multidimensional assessment of adverse events of finasteride：a real-world pharmacovigilance analysis based on FDA Adverse Event Reporting System (FAERS) from 2004 to April 2024
Source: PLoS One. 2025 Mar 24;20(3):e0309849. doi: 10.1371/journal.pone.0309849 (PMC11932486; doi:10.1371/journal.pone.0309849)
Supplement: Peer review history

# Author comment: Author response to Decision Letter 0

**DOI:** 10.1371/journal.pone.0309849.r001

18 Aug 2024

Attachment Submitted filename: PLOSOne Human Subjects Research Checklist.docx

# Peer review: Decision Letter 0

**DOI:** 10.1371/journal.pone.0309849.r002

**Authors:** Roland Eghoghosoa Akhigbe

1 Dec 2024

PONE-D-24-35263Multidimensional assessment of adverse events of finasteride:a real-world pharmacovigilance analysis based on FDA Adverse Event Reporting System (FAERS) from 2004 to April 2024PLOS ONE

Dear Dr. Liu,

Thank you for submitting your manuscript to PLOS ONE. After careful consideration, we feel that it has merit but does not fully meet PLOS ONE’s publication criteria as it currently stands. Therefore, we invite you to submit a revised version of the manuscript that addresses the points raised during the review process.

Please submit your revised manuscript by Jan 15 2025 11:59PM. If you will need more time than this to complete your revisions, please reply to this message or contact the journal office at plosone@plos.org . When you're ready to submit your revision, log on to https://www.editorialmanager.com/pone/ and select the 'Submissions Needing Revision' folder to locate your manuscript file.

Please include the following items when submitting your revised manuscript:

- A rebuttal letter that responds to each point raised by the academic editor and reviewer(s). You should upload this letter as a separate file labeled 'Response to Reviewers'.
- A marked-up copy of your manuscript that highlights changes made to the original version. You should upload this as a separate file labeled 'Revised Manuscript with Track Changes'.
- An unmarked version of your revised paper without tracked changes. You should upload this as a separate file labeled 'Manuscript'.

If you would like to make changes to your financial disclosure, please include your updated statement in your cover letter. Guidelines for resubmitting your figure files are available below the reviewer comments at the end of this letter.

If applicable, we recommend that you deposit your laboratory protocols in protocols.io to enhance the reproducibility of your results. Protocols.io assigns your protocol its own identifier (DOI) so that it can be cited independently in the future. For instructions see: https://journals.plos.org/plosone/s/submission-guidelines#loc-laboratory-protocols . Additionally, PLOS ONE offers an option for publishing peer-reviewed Lab Protocol articles, which describe protocols hosted on protocols.io. Read more information on sharing protocols at https://plos.org/protocols?utm_medium=editorial-email&utm_source=authorletters&utm_campaign=protocols .

We look forward to receiving your revised manuscript.

Kind regards,

Roland Eghoghosoa Akhigbe

Academic Editor

PLOS ONE

Journal requirements:

When submitting your revision, we need you to address these additional requirements.

1.  Please ensure that your manuscript meets PLOS ONE's style requirements, including those for file naming. The PLOS ONE style templates can be found at

https://journals.plos.org/plosone/s/file?id=wjVg/PLOSOne_formatting_sample_main_body.pdf   and

https://journals.plos.org/plosone/s/file?id=ba62/PLOSOne_formatting_sample_title_authors_affiliations.pdf

2. We note that Figure 2 in your submission contain [map/satellite] images which may be copyrighted. All PLOS content is published under the Creative Commons Attribution License (CC BY 4.0), which means that the manuscript, images, and Supporting Information files will be freely available online, and any third party is permitted to access, download, copy, distribute, and use these materials in any way, even commercially, with proper attribution. For these reasons, we cannot publish previously copyrighted maps or satellite images created using proprietary data, such as Google software (Google Maps, Street View, and Earth). For more information, see our copyright guidelines: http://journals.plos.org/plosone/s/licenses-and-copyright.

We require you to either (1) present written permission from the copyright holder to publish these figures specifically under the CC BY 4.0 license, or (2) remove the figures from your submission:

a. You may seek permission from the original copyright holder of Figure 2 to publish the content specifically under the CC BY 4.0 license. 

We recommend that you contact the original copyright holder with the Content Permission Form (http://journals.plos.org/plosone/s/file?id=7c09/content-permission-form.pdf) and the following text:

“I request permission for the open-access journal PLOS ONE to publish XXX under the Creative Commons Attribution License (CCAL) CC BY 4.0 (http://creativecommons.org/licenses/by/4.0/). Please be aware that this license allows unrestricted use and distribution, even commercially, by third parties. Please reply and provide explicit written permission to publish XXX under a CC BY license and complete the attached form.”

Please upload the completed Content Permission Form or other proof of granted permissions as an ""Other"" file with your submission.

In the figure caption of the copyrighted figure, please include the following text: “Reprinted from [ref] under a CC BY license, with permission from [name of publisher], original copyright [original copyright year].”

b. If you are unable to obtain permission from the original copyright holder to publish these figures under the CC BY 4.0 license or if the copyright holder’s requirements are incompatible with the CC BY 4.0 license, please either i) remove the figure or ii) supply a replacement figure that complies with the CC BY 4.0 license. Please check copyright information on all replacement figures and update the figure caption with source information. If applicable, please specify in the figure caption text when a figure is similar but not identical to the original image and is therefore for illustrative purposes only.

The following resources for replacing copyrighted map figures may be helpful:

USGS National Map Viewer (public domain): http://viewer.nationalmap.gov/viewer/

The Gateway to Astronaut Photography of Earth (public domain): http://eol.jsc.nasa.gov/sseop/clickmap/

Maps at the CIA (public domain): https://www.cia.gov/library/publications/the-world-factbook/index.html and https://www.cia.gov/library/publications/cia-maps-publications/index.html

NASA Earth Observatory (public domain): http://earthobservatory.nasa.gov/

Landsat: http://landsat.visibleearth.nasa.gov/

USGS EROS (Earth Resources Observatory and Science (EROS) Center) (public domain): http://eros.usgs.gov/#

Natural Earth (public domain): http://www.naturalearthdata.com/

3. Please include your tables as part of your main manuscript and remove the individual files. Please note that supplementary tables (should remain/ be uploaded) as separate "supporting information" files

4. Please review your reference list to ensure that it is complete and correct. If you have cited papers that have been retracted, please include the rationale for doing so in the manuscript text, or remove these references and replace them with relevant current references. Any changes to the reference list should be mentioned in the rebuttal letter that accompanies your revised manuscript. If you need to cite a retracted article, indicate the article’s retracted status in the References list and also include a citation and full reference for the retraction notice.

[Note: HTML markup is below. Please do not edit.]

Reviewers' comments:

Reviewer's Responses to Questions

**Comments to the Author**

1. Is the manuscript technically sound, and do the data support the conclusions?

The manuscript must describe a technically sound piece of scientific research with data that supports the conclusions. Experiments must have been conducted rigorously, with appropriate controls, replication, and sample sizes. The conclusions must be drawn appropriately based on the data presented. 

Reviewer #1: Yes

Reviewer #2: Yes

**********

2. Has the statistical analysis been performed appropriately and rigorously? 

Reviewer #1: I Don't Know

Reviewer #2: Yes

**********

3. Have the authors made all data underlying the findings in their manuscript fully available?

The PLOS Data policy requires authors to make all data underlying the findings described in their manuscript fully available without restriction, with rare exception (please refer to the Data Availability Statement in the manuscript PDF file). The data should be provided as part of the manuscript or its supporting information, or deposited to a public repository. For example, in addition to summary statistics, the data points behind means, medians and variance measures should be available. If there are restrictions on publicly sharing data—e.g. participant privacy or use of data from a third party—those must be specified.

Reviewer #1: Yes

Reviewer #2: Yes

**********

4. Is the manuscript presented in an intelligible fashion and written in standard English?

PLOS ONE does not copyedit accepted manuscripts, so the language in submitted articles must be clear, correct, and unambiguous. Any typographical or grammatical errors should be corrected at revision, so please note any specific errors here.

Reviewer #1: Yes

Reviewer #2: Yes

**********

5. Review Comments to the Author

Please use the space provided to explain your answers to the questions above. You may also include additional comments for the author, including concerns about dual publication, research ethics, or publication ethics. (Please upload your review as an attachment if it exceeds 20,000 characters)

Reviewer #1: Zhong and colleagues use statistical approaches to categorize and analyze spontaneous adverse events (AEs) for the widely used hair loss medicine finasteride in the last 20 years using the publicly available FAERS database. This work aims to evaluate the common adverse events for finasteride better and help prioritize safety precautions and research in the correct direction. I suggest this paper to be published with minor revisions of the points I will list below:

Scientific suggestions:

1) The authors use four statistical tools to detect the suspected AEs of finasteride use using the real-life patient reports. In their analysis, an AE signal that meets the criteria for any of these four methods is considered as a drug-associated AE. However, these algorithms have multiple strengths and weaknesses over each other, and AEs that have highest overlap of signals would be more reliable to identify as finasteride associated. To reflect the overlap and differences between these methods, a heatmap of the signals of AEs detected by ROR, PRR, BCPNN, and MGPS methods at the SOC level will be useful to include.

2) The authors state their suggestions for finasteride use and further investigation of the biology of this drug based on their analysis of patient AE reports. Although, these are insightful findings and recommendations, it is important to mention the biases in these datasets. For instance, in the Discussion section, the authors suggest close monitoring for potential risks of this drug over a month based on the high number of reports in this time period. However, patients will tend to link and report side effects that they experience in a short time after they start to use the drug, resulting in a biased dataset.

3) Since the study focuses on the biological differences of patients using “sex” instead of “gender” is more appropriate. The authors use these words interchangeably through the text.

Points that need to be addressed in writing for the publication:

4) There are repetitive and incomplete sentences that must be fixed.

Examples include:

- “These methods were used to evaluate the of finasteride medication and AEs. whether there is a significant association between finasteride drug use and AEs.”

- “Finally, by means of the Medex UIMA 1.8.3 system the standardization of drug

names[21,22]. Finally, by means of the Medex UIMA 1.8.3 system the standardization of drug names.”

- “This is probably due to the fact that the younger patients have a more stressful life and are more sensitive to hormonal changes than the older ones[39]. In addition, in the PT analysis we observed ‘sexual dysfunction’ and ‘catastrophic reaction’. This may be due to the fact that younger patients have more stressful lives, are more sensitive to hormonal changes than older adults, are more prone to symptoms, and are more susceptible to the adverse effects of finasteride.”

5) Abbreviations should be spelled out in the text where they are used the first time such as Benign prostatic hyperplasia (BPH).

6) Word missing: “These methods were used to evaluate the XXX of finasteride medication and AEs.”

7) Space missing: “Emergingevidence”

8) PT should be capitalized: “… the only pt’s with positive signals …”

Reviewer #2: 1. Adverse effects should be summarized in tabulated form alongwith their relation to demographics & external factors

2. Duration of treatment with demographics should be statistically analyzed

3. Doses of the drug should be mentioned at which ADRs appeared

4. How anxiety & depression was analyzed? Which scale was used?

5. Make the findings more summarized

**********

6. PLOS authors have the option to publish the peer review history of their article (what does this mean? ). If published, this will include your full peer review and any attached files.

If you choose “no”, your identity will remain anonymous but your review may still be made public.

**Do you want your identity to be public for this peer review?** For information about this choice, including consent withdrawal, please see our Privacy Policy .

Reviewer #1: No

Reviewer #2: No

**********

[NOTE: If reviewer comments were submitted as an attachment file, they will be attached to this email and accessible via the submission site. Please log into your account, locate the manuscript record, and check for the action link "View Attachments". If this link does not appear, there are no attachment files.]

While revising your submission, please upload your figure files to the Preflight Analysis and Conversion Engine (PACE) digital diagnostic tool, https://pacev2.apexcovantage.com/ . PACE helps ensure that figures meet PLOS requirements. To use PACE, you must first register as a user. Registration is free. Then, login and navigate to the UPLOAD tab, where you will find detailed instructions on how to use the tool. If you encounter any issues or have any questions when using PACE, please email PLOS at figures@plos.org . Please note that Supporting Information files do not need this step.

# Author comment: Author response to Decision Letter 1

**DOI:** 10.1371/journal.pone.0309849.r003

7 Jan 2025

Journals' comments:

Comment 1: Please ensure that your manuscript meets PLOS ONE's style requirements, including those for file naming.

Response 1: Thank you for your suggestion. I have carefully reviewed the manuscript and ensured that it complies with PLOS ONE's style requirements, including those related to file naming.

Comment 2: We note that Figure 2 in your submission contain [map/satellite] images which may be copyrighted.

Response 2: Thank you for your valuable feedback. According to the journal's map copyright guidelines, "If you created the map in a software program like R or ArcGIS, please locate the source of the basemap within the package used to generate the map." Figure 2 was created using the mapdata package in R software, adding the basemap source (https://cran.r-project.org/web/packages/mapdata/index.html) to the manuscript.

Page 12, Lines 309-311.

Comment 3: Please include your tables as part of your main manuscript and remove the individual files. Please note that supplementary tables (should remain/ be uploaded) as separate "supporting information" files.

Response 3: Thank you for your suggestion. We have incorporated Table 1 and Table 2 into the main manuscript and removed the individual files. The supplementary tables have been uploaded as separate "supporting information" files as requested.

Comment 4: Please review your reference list to ensure that it is complete and correct. If you have cited papers that have been retracted, please include the rationale for doing so in the manuscript text, or remove these references and replace them with relevant current references. Any changes to the reference list should be mentioned in the rebuttal letter that accompanies your revised manuscript. If you need to cite a retracted article, indicate the article’s retracted status in the References list and also include a citation and full reference for the retraction notice.

Response 4: Thank you for your thorough feedback. We have carefully reviewed the reference list to ensure it is complete and accurate. Each reference has been checked individually, and we confirm that no retracted papers have been cited.

Comment 5: While revising your submission, please upload your figure files to the Preflight Analysis and Conversion Engine (PACE) digital diagnostic tool, https://pacev2.apexcovantage.com/.

Response 5：Thank you for your suggestion, the chart file has been uploaded to the Pre-Check Analysis and Conversion Engine (PACE) digital diagnostic tool for proofreading.

Reviewers' comments:

Reviewer #1:

Comment 1: The authors use four statistical tools to detect the suspected AEs of finasteride use using the real-life patient reports. In their analysis, an AE signal that meets the criteria for any of these four methods is considered as a drug-associated AE. However, these algorithms have multiple strengths and weaknesses over each other, and AEs that have highest overlap of signals would be more reliable to identify as finasteride associated. To reflect the overlap and differences between these methods, a heatmap of the signals of AEs detected by ROR, PRR, BCPNN, and MGPS methods at the SOC level will be useful to include.

Response 1: Thank you for your valuable suggestion. In response, we have added Figure 3, which presents a heatmap of AE signals detected by the ROR, PRR, BCPNN, and MGPS methods at the SOC level. This provides a more intuitive visualization of the data presented in S3_Table.

Page 13, Line 328.

Comment 2: The authors state their suggestions for finasteride use and further investigation of the biology of this drug based on their analysis of patient AE reports. Although, these are insightful findings and recommendations, it is important to mention the biases in these datasets. For instance, in the Discussion section, the authors suggest close monitoring for potential risks of this drug over a month based on the high number of reports in this time period. However, patients will tend to link and report side effects that they experience in a short time after they start to use the drug, resulting in a biased dataset.

Response 2: Thank you for your insightful comment. In response, we have addressed this concern in the Discussion section by adding the statement: “However, it is also possible that patients linked and reported their medication side effects within a short period of time after using the drug, which could also lead to bias in the data set.”

Page 28, Lines 707–709.

Comment 3: Since the study focuses on the biological differences of patients using “sex” instead of “gender” is more appropriate. The authors use these words interchangeably through the text.

Response 3: Thank you for your valuable suggestion. We have carefully revised the manuscript to use “sex” instead of “gender” throughout the text to ensure appropriate terminology is used.

Comment 4: There are repetitive and incomplete sentences that must be fixed.

Examples include:

- “These methods were used to evaluate the of finasteride medication and AEs. whether there is a significant association between finasteride drug use and AEs.”

- “Finally, by means of the Medex UIMA 1.8.3 system the standardization of drug

names[21,22]. Finally, by means of the Medex UIMA 1.8.3 system the standardization of drug names.”

- “This is probably due to the fact that the younger patients have a more stressful life and are more sensitive to hormonal changes than the older ones[39]. In addition, in the PT analysis we observed ‘sexual dysfunction’ and ‘catastrophic reaction’. This may be due to the fact that younger patients have more stressful lives, are more sensitive to hormonal changes than older adults, are more prone to symptoms, and are more susceptible to the adverse effects of finasteride.”

Response 4:

Thank you for your valuable feedback. We have addressed the repetitive and incomplete sentences and checked that there are no similar errors elsewhere in the full manuscript, as follows:

�The repetitive phrase “the of finasteride medication and AEs” has been deleted.

Page 2, Line 41.

�The redundant sentence “Finally, by means of the Medex UIMA 1.8.3 system the standardization of drug names” has been removed.

Page 6, Line 181.

�The duplicated sentences “This is probably due to the fact that the younger patients have a more stressful life and are more sensitive to hormonal changes than the older ones. In addition, in the PT analysis we observed ‘sexual dysfunction’ and ‘catastrophic reaction’. This may be due to the fact that younger patients have more stressful lives, are more sensitive to hormonal changes than older adults” have been removed.

Page 23, Line 561

�As per your suggestion, we have made revisions to other repetitive sections of the manuscript. Specifically, the sentence "After excluding inaccurate, missing, or unknown gender time of onset reports, a total of 7019 finasteride-administered AEs reported time of onset." has been removed.

Page 20, Lines 420.

Comment 5: Abbreviations should be spelled out in the text where they are used the first time such as Benign prostatic hyperplasia (BPH).

Response 5: Thank you for your valuable suggestion. We have revised the manuscript to ensure that abbreviations are spelled out in full the first time they appear in the text, such as “Benign prostatic hyperplasia (BPH)”.

Page 4, Lines 110–111.

Comment 6: Word missing: “These methods were used to evaluate the XXX of finasteride medication and AEs.”

Response 6: Thank you for your helpful feedback. We have corrected the expression by removing the phrase “the of finasteride medication and AEs”.

Page 2, Line 41.

Comment 7: Space missing: “Emergingevidence”

Response7：Thank you for your careful observation. We have corrected the phrase “Emergingevidence” to “Emerging evidence”.

Page 25, Line 625.

Comment 8: PT should be capitalized: “… the only pt’s with positive signals …”

Response 8: Thank you for your valuable feedback. We have made the necessary correction and changed “pt’s” to “PT’s”.

Page 25, Line 627.

Reviewer #2: 

Comment 1: Adverse effects should be summarized in tabulated form alongwith their relation to demographics & external factors

Response 1: Thank you for your insightful suggestion. In response, we have summarized the adverse effects in relation to demographics and external factors. The gender differences are presented in Figure 5 as a heatmap, and the relationship between adverse effects and age subgroups has been added to S9_Table.

We fully agree with the reviewers on the importance of considering external factors, such as environmental or genetic influences, when analyzing adverse effects. However, as the database used for this study only provides limited demographic data (including year, sex, age, reporter, reported countries, route, outcomes, time to onset, and indications, as shown in Table 1), we acknowledge that our analysis of these external factors is constrained. Despite these limitations, we have endeavored to extract as much valuable information as possible from the available data, ensuring a thorough analysis aligned with the study's primary objectives.

Page 22, Line 511

Page 45, Line 1116-1117

Comment 2: Duration of treatment with demographics should be statistically analyzed

Response 2: Thank you for your valuable suggestion. While we understand the importance of analyzing the duration of treatment along with demographics, we must note that the FAERS data used in this study is primarily focused on drug safety signal analysis. Unfortunately, the data regarding treatment duration is often incomplete or unreliable, as some reports lack specific dates, particularly the end date of treatment. Given these limitations, we feel that conducting a detailed statistical analysis of treatment duration in this context may not provide accurate insights. We believe that further investigation into the role of treatment duration would be better suited for clinical trial data.We explain this in the Limitation section.

We appreciate your understanding and thoughtful feedback.

Page 29, Line 769-773

Comment 3: Doses of the drug should be mentioned at which ADRs appeared

Response 3: Thank you for your helpful suggestion. We have added the drug dose at which the ADRs appeared, specifying “1mg once daily”, and have included the relevant reference [1].

Page 24, Line 597-599

Page 37, Lines 951-952

Comment 4: How anxiety & depression was analyzed? Which scale was used?

Response 4: Thank you for your enlightening question. Unfortunately, the FAERS database (https://open.fda.gov/data/downloads/) does not include specific scales for anxiety and depression, so this information is not available. In addition, the primary focus of this study is on the adverse events (AEs) reported or not reported in the finasteride drug label and the relationship between AEs and age and sex.

As a comprehensive sensitivity analysis was conducted in a 2021 JAMA study that found no causal association between androgenetic alopecia and psychological adverse events in younger individuals, we did not pursue an in-depth analysis of this issue. We have added this clarification in the Discussion section and included the relevant reference [2].

Page 24, lines 615-627

Page 39, lines 983-985

Comment 5: Make the findings more summarized

Response 5: Thank you for your helpful suggestion. In response, we have revised the Results section to make the findings more concise and summarized. The relevant sections have been updated as follows:

Page 2, Lines 46–62

Page 9, Lines 254–271

Page 13, Lines 316–339

Page 13, Lines 341–346

Page 21, Lines 454–461

Page 22, Lines 466–542

[1] Devjani S, Ezemma O, Kelley KJ, Stratton E, Senna M. Androgenetic Alopecia: Therapy Update. Drugs. 2023;83: 701–715. doi:10.1007/s40265-023-01880-x

[2] Choi JW, Huh CH, Choi GS. Association of Hair Loss With Suicidality and Psychological Adverse Events vs Finasteride Use. JAMA Dermatol. 2021;157: 737. doi:10.1001/jamadermatol.2021.1001

Attachment Submitted filename: Response to Reviewers.docx

# Peer review: Decision Letter 1

**DOI:** 10.1371/journal.pone.0309849.r004

**Authors:** Roland Eghoghosoa Akhigbe

13 Feb 2025

Multidimensional assessment of adverse events of finasteride:a real-world pharmacovigilance analysis based on FDA Adverse Event Reporting System (FAERS) from 2004 to April 2024

PONE-D-24-35263R1

Dear Dr. Liu,

We’re pleased to inform you that your manuscript has been judged scientifically suitable for publication and will be formally accepted for publication once it meets all outstanding technical requirements.

Within one week, you’ll receive an e-mail detailing the required amendments. When these have been addressed, you’ll receive a formal acceptance letter and your manuscript will be scheduled for publication.

An invoice will be generated when your article is formally accepted. Please note, if your institution has a publishing partnership with PLOS and your article meets the relevant criteria, all or part of your publication costs will be covered. Please make sure your user information is up-to-date by logging into Editorial Manager at Editorial Manager®  and clicking the ‘Update My Information' link at the top of the page. If you have any questions relating to publication charges, please contact our Author Billing department directly at authorbilling@plos.org.

If your institution or institutions have a press office, please notify them about your upcoming paper to help maximize its impact. If they’ll be preparing press materials, please inform our press team as soon as possible -- no later than 48 hours after receiving the formal acceptance. Your manuscript will remain under strict press embargo until 2 pm Eastern Time on the date of publication. For more information, please contact onepress@plos.org.

Kind regards,

Roland Eghoghosoa Akhigbe

Academic Editor

PLOS ONE

Additional Editor Comments (optional): The reviewers are now satisfied with the manuscript and have recommended that it be accepted for publication. I have also gone through the revised version and found it suitable for publication. Congratulations. 

Reviewers' comments:

Reviewer's Responses to Questions

**Comments to the Author**

1. If the authors have adequately addressed your comments raised in a previous round of review and you feel that this manuscript is now acceptable for publication, you may indicate that here to bypass the “Comments to the Author” section, enter your conflict of interest statement in the “Confidential to Editor” section, and submit your "Accept" recommendation.

Reviewer #1: All comments have been addressed

Reviewer #2: All comments have been addressed

**********

2. Is the manuscript technically sound, and do the data support the conclusions?

The manuscript must describe a technically sound piece of scientific research with data that supports the conclusions. Experiments must have been conducted rigorously, with appropriate controls, replication, and sample sizes. The conclusions must be drawn appropriately based on the data presented. 

Reviewer #1: Yes

Reviewer #2: Yes

**********

3. Has the statistical analysis been performed appropriately and rigorously? 

Reviewer #1: I Don't Know

Reviewer #2: Yes

**********

4. Have the authors made all data underlying the findings in their manuscript fully available?

The PLOS Data policy requires authors to make all data underlying the findings described in their manuscript fully available without restriction, with rare exception (please refer to the Data Availability Statement in the manuscript PDF file). The data should be provided as part of the manuscript or its supporting information, or deposited to a public repository. For example, in addition to summary statistics, the data points behind means, medians and variance measures should be available. If there are restrictions on publicly sharing data—e.g. participant privacy or use of data from a third party—those must be specified.

Reviewer #1: Yes

Reviewer #2: Yes

**********

5. Is the manuscript presented in an intelligible fashion and written in standard English?

PLOS ONE does not copyedit accepted manuscripts, so the language in submitted articles must be clear, correct, and unambiguous. Any typographical or grammatical errors should be corrected at revision, so please note any specific errors here.

Reviewer #1: Yes

Reviewer #2: Yes

**********

6. Review Comments to the Author

Please use the space provided to explain your answers to the questions above. You may also include additional comments for the author, including concerns about dual publication, research ethics, or publication ethics. (Please upload your review as an attachment if it exceeds 20,000 characters)

Reviewer #1: The authors addressed the critical issues that were brought up in the first submission. Revised version is fit for publication.

Reviewer #2: (No Response)

**********

7. PLOS authors have the option to publish the peer review history of their article (what does this mean? ). If published, this will include your full peer review and any attached files.

If you choose “no”, your identity will remain anonymous but your review may still be made public.

**Do you want your identity to be public for this peer review?** For information about this choice, including consent withdrawal, please see our Privacy Policy .

Reviewer #1: No

Reviewer #2: No

**********

# Editor report: Acceptance letter

**DOI:** 10.1371/journal.pone.0309849.r005

**Authors:** Roland Eghoghosoa Akhigbe

PONE-D-24-35263R1

PLOS ONE

Dear Dr. Liu,

I'm pleased to inform you that your manuscript has been deemed suitable for publication in PLOS ONE. Congratulations! Your manuscript is now being handed over to our production team.

At this stage, our production department will prepare your paper for publication. This includes ensuring the following:

* All references, tables, and figures are properly cited

* All relevant supporting information is included in the manuscript submission,

* There are no issues that prevent the paper from being properly typeset

If revisions are needed, the production department will contact you directly to resolve them. If no revisions are needed, you will receive an email when the publication date has been set. At this time, we do not offer pre-publication proofs to authors during production of the accepted work. Please keep in mind that we are working through a large volume of accepted articles, so please give us a few weeks to review your paper and let you know the next and final steps.

Lastly, if your institution or institutions have a press office, please let them know about your upcoming paper now to help maximize its impact. If they'll be preparing press materials, please inform our press team within the next 48 hours. Your manuscript will remain under strict press embargo until 2 pm Eastern Time on the date of publication. For more information, please contact onepress@plos.org.

If we can help with anything else, please email us at customercare@plos.org.

Thank you for submitting your work to PLOS ONE and supporting open access.

Kind regards,

PLOS ONE Editorial Office Staff

on behalf of

Dr. Roland Eghoghosoa Akhigbe

Academic Editor

PLOS ONE

Attachment Submitted filename: pone.0309849.docx
